# Digital Interventions to Reduce Sedentary Behaviors of Office Workers: Scoping Review

**DOI:** 10.2196/11079

**Published:** 2019-02-07

**Authors:** Yitong Huang, Steve Benford, Holly Blake

**Affiliations:** 1 Horizon Centre for Doctoral Training University of Nottingham Nottingham United Kingdom; 2 Mixed Reality Laboratory School of Computer Science University of Nottingham Nottingham United Kingdom; 3 School of Health Sciences University of Nottingham Nottingham United Kingdom; 4 National Institute for Health Research Nottingham Biomedical Research Centre Nottingham United Kingdom

**Keywords:** telemedicine, sedentary behavior, workplace, technology, internet, microcomputers

## Abstract

**Background:**

There is a clear public health need to reduce office workers’ sedentary behaviors (SBs), especially in the workplace. Digital technologies are increasingly being deployed in the workplace to measure and modify office workers’ SBs. However, knowledge of the range and nature of research on this topic is limited; it also remains unclear to what extent digital interventions have exploited the technological possibilities.

**Objective:**

This study aimed to investigate the technological landscape of digital interventions for SB reduction in office workers and to map the research activity in this field.

**Methods:**

Terms related to SB, office worker, and digital technology were applied in various combinations to search Cochrane Library, Joanna Briggs Institute Database of Systematic Reviews, MEDLINE, PsycARTICLES, PsycINFO, Scopus, Association for Computing Machinery Digital Library, Engineering index Compendex, and Google Scholar for the years 2000 to 2017. Data regarding the study and intervention details were extracted. Interventions and studies were categorized into development, feasibility and/piloting, evaluation, or implementation phase based on the UK Medical Research Council (MRC) framework for developing and evaluating complex interventions. A novel framework was developed to classify technological features and annotate technological configurations. A mix of quantitative and qualitative approaches was used to summarize data.

**Results:**

We identified 68 articles describing 45 digital interventions designed to intervene with office workers’ SB. A total of 6 common technological features had been applied to interventions with various combinations. Configurations such as “information delivery and mediated organizational and social support” and “digital log and automated tailored feedback” were well established in evaluation and implementation studies; in contrast, the integration of passive data collection, connected devices, and ATF or scheduled prompts was mostly present in development and piloting research.

**Conclusions:**

This review is the first to map and describe the use of digital technologies in research on SB reduction in office workers. Interdisciplinary collaborations can help to maximize the potential of technologies. As novel modes of delivery that capitalize on embedded computing and electronics, wireless technologies have been developed and piloted in engineering, computing, and design fields, efforts can be directed to move them to the next phase of evaluation with more rigorous study designs. Quality of research may be improved by fostering conversations between different research communities and encouraging researchers to plan, conduct, and report their research under the MRC framework. This review will be particularly informative to those deciding on areas where further research or development is needed and to those looking to locate the relevant expertise, resources, and design inputs when designing their own systems or interventions.

## Introduction

### Background

Sedentary behaviors (SBs) are activities that require very low energy expenditure of less than 1.5 metabolic equivalents and typically involve lying down and sitting [1]. Excessive SB is recognized as an exposure to a risk factor different from a lack of moderate to vigorous physical activity (MVPA), as an individual who engages in 150 min of exercise every week can still spend the majority of the remaining waking hours in SB. Reducing SB may require approaches very different from those required to increase physical activity (PA), as sedentary time can accumulate unintentionally in a broad range of contexts such as during leisure time, transportation, and in the workplace. Although a recent meta-analysis [2]indicates that 60 to 75 min of MVPA per day seems to offset the increased risks of mortality associated with sitting for more than 8 hours per day, this amount of MVPA is notably beyond the recommended levels of MVPA in most public health guidelines [3,4]. More importantly, mounting evidence suggests reducing SB, especially prolonged episodes of SB, has its own benefits on metabolic and musculoskeletal health, and potentially on other health conditions [5-8].

A number of studies [9-11] have found that office workers’ within-work time is characterized by more prolonged SB with fewer breaks than nonwork time; sedentary work contributes significantly to overall sedentary exposure of office workers and, thus, the health risks. A recent statement by an international panel of experts highlighted the need for interventions that target the reduction of prolonged SB in this setting and population, for both better health and productivity outcomes [12]. In this paper, we focus on a potential solution: SB interventions delivered with digital technologies.

According to the Oxford Dictionary, digital technologies refer to technologies involving or relating to the use of computer technology, which includes tools, systems, devices, and resources that generate, store, or process data in the form of digital signals. The past decades have seen an exponential growth of computing power at affordable prices. This has resulted in an increasing variety of digital gadgets (eg, personal computer, tablets, smartphones, wearables, and Internet of Things) that a person is exposed to and interacts with on a day-to-day basis. This presents health intervention designers and researchers with a wider range of device choices that offer different form factors and features. Indeed, digital health has demonstrated great promise in a range of clinical settings and populations in terms of behavioral measurement and intervention delivery (eg, pediatric care [13] and mental health [14]).

However, when it comes to digital SB interventions, the behavioral target of “being less sedentary” and the use of digital media seem to present us with a paradox here. First, the increase in sedentary occupations and sedentariness at work in itself is closely related to the evolution of digital technology, which enables more work to be completed at desks without manual labor or even light PA. Second, a recent study [15] has found that information and communication technologies (ICTs) have supported new break activities in the office (eg, checking social media during mini-breaks or watching videos over lunch breaks) while evoking negative feelings at the same time. The researchers used the term “screen guilt” to describe office workers’ need to disconnect from screen-based ICTs during breaks for both physical and psychological well-being.

This has led us to rethink what (or even whether) digital features should be incorporated when designing SB interventions. The intersection of digital health and SB has attracted a lot of research interest and accumulated a large body of interdisciplinary research in recent years. As a first step in our own research on exploiting novel digital technologies for the delivery of workplace SB interventions, we wanted to review the literature on this topic in a systematic manner, to map the current technological landscape and research activities conducted in different disciplines, and to determine research gaps in terms of utilizing and innovating technologies for workplace SB interventions.

### Previous Reviews

To date, 7 systematic reviews on SB interventions targeting adults have been published [16-22]. This section overviews which aspects of the topic have been addressed in those reviews and which areas require more secondary research.

All the reviews were inclusive of all SB reduction interventions regardless of the presence of digital elements. Chau and colleagues [16] reviewed workplace studies published up to April 2009 and identified only 6 eligible studies that included sitting as an outcome measure. Only 2 types of digital media were covered (emails [23-25] and pedometer [23]). Measurement of SB was self-reported in all 6 studies, none of which found significant intervention effect on sitting reduction. The result was inconclusive with respect to the most appropriate intervention approach or delivery mode because of disparate study designs and delivery modes across studies. With a similar inclusion criterion as Chau and colleagues’ [16], a more recent review [17] by Shrestha and colleagues identified 20 eligible workplace studies published up to June 2015. The analysis was focused on comparing the effects of different intervention components with absence of these components or alternative components. Only a small part of the analysis was pertinent to digital interventions. First, it compared the effect of computer prompts plus information counseling on sitting reduction with information counseling only, based on data from 3 studies [26-28]. Second, it compared the effect of different contents in e-newsletters on sitting reduction, based on 1 study [29]. The findings from both analyses were nonsignificant or inconclusive, given the low quality of evidence. Commissaris and colleagues [18] specifically reviewed workplace SB interventions aimed to influence workers’ SB while doing productive work. As a small part of their analyses, they compared 6 interventions including self-monitoring of SB and/or PA (using devices such as pedometers) with 4 interventions not including self-monitoring and suggested that self-monitoring seemed to be ineffective in improving SB/PA at work. Another review of workplace SB interventions by Chu and colleagues [19] included 26 studies published up to December 2015 and classified them based on intervention strategies into 3 categories: (1) environmental strategies, (2) educational/ behavioral strategies (involving educational program and point-of-choice motivational signs), and (3) combined strategies. They concluded from subgroup analyses that interventions combining multiple components resulted in the greatest sitting reduction, followed by environmental strategies. However, the review did not distinguish digital and nondigital delivery of intervention strategies within each category. Similar to Chu and colleagues’ review [19], Gardener and colleagues’ review [30] was also focused on intervention strategies, but with a broader scope (ie, including nonworkplace studies) and a more fine-grained coding scheme based on the underlying intervention functions [31] and behavior change techniques (BCTs) [32]. They found that education, persuasion, environmental restructuring, and training were the most promising intervention functions and that self-monitoring, problem solving, and changing the social or physical environment were particularly promising BCTs for reducing SB. Martin and colleagues’ review [21] was also inclusive of nonworkplace interventions. It was suggested that interventions targeting SB only and lifestyle change might be more promising than those targeting PA only or a combination of PA and SB, which was similar to the conclusion reached in Prince and colleagues’ review [22].

Although shedding light on intervention strategies and components effective for reducing workplace SB, those reviews fell short in 2 aspects.

First, they did not differentiate diverse ways an intervention strategy/component could be digitally implemented and delivered. For instance, for the same strategy of point-of-choice prompts, the actual quantities of prompts received and noticed by participants may differ significantly depending on whether the break reminder was delivered on workstation screens, by smartphone notifications, or via tactile feedback from wearable devices. Apart from specific technological features, how different features were applied in combination and in support of each other is also worthy of attention. For instance, just-in-time adaptive intervention (JITAI), an approach that employs context-aware sensing and computing to detect the behavioral context and tailor the intervention in real time, can address the dynamically changing needs of individuals much better than a traditional intervention delivering static content with a fixed schedule [33]. Knowledge of such nuances in technological design is important as they may lead to considerable difference in the quality and quantity of interventions delivered to participants, making outcomes incomparable across studies.

Second, none of the abovementioned reviews included the engineering and computer science literature, despite the rapid prototyping and piloting of novel technologies within these fields that may become or inform the next generation of digital interventions. An exploratory search of this body of literature has found an abundance of user-centered design research [34] on technologies targeting SB reduction in office workers. Those studies, although employing very different study designs from clinical trials, have gathered valuable data about design-related outcomes (technological feasibility, usability, and acceptability) usually by involving stakeholders from the outset of intervention development. The findings do not only inform technology design but also give an indication of the potential user uptake, attitude, and adherence to different intervention technologies should they be moved to later stages of development and evaluation. As yet, awareness of the size and location of this body of evidence is lacking.

### This Review

In summary, although previous reviews have touched on the technological design in SB interventions, there is a need for a review that is dedicated to this topic and that encompasses a wider range of literature. Specifically, the following questions can be explored:

1. How have digital technologies been used in interventions to reduce office workers’ SB at work?
2. What research has been done on them and what development phases have they reached?
3. Where does the research gap lie as to utilizing and innovating digital technologies for SB interventions targeting office workers?

In view of the above, we selected the approach of scoping review, which is a particularly useful tool to synthesize findings established with different study designs and to address broader topics than those addressed by systematic reviews (eg, effectiveness) [35].

The review will be reported with the following structure. Considering the complexity of this topic, we will first review existing classifications and frameworks proposed from several disciplines to describe digital technologies for behavior change. Second, we present the search and review method. In the Results section, we first provide a quantitative summary of studies and interventions identified in this review. Then, we narratively overview the range of research conducted on interventions with different technological designs and summarize the findings pertinent to the technological features. Finally, we discuss findings and suggest avenues for future research.

This review is not aimed to estimate the efficacy of interventions with or without certain digital components, which should be addressed by further systematic reviews once the technological landscape is laid out. Neither is this review focused on comparing the capabilities and limitations of various brands of technological devices, which have been featured in other studies [36,37]. Instead, the main objective here is to scope research across different fields through review of the technological features present in interventions and mapping different research activities (eg, design-led research, feasibility studies, and experimental studies) onto different stages in the process of intervention development and evaluation. Another objective is to synthesize the design-related findings (eg, satisfaction, usability, acceptability, feasibility, and engagement) of digital interventions, which were overlooked in previous reviews.

### Existing Frameworks and Classifications for Digital Health Technologies

The technological aspect of digital health has been discussed under several umbrella terms such as persuasive technology (PT)/system [38,39], behavioral intervention technology (BIT) [40], and mode of delivery (MoD) for behavior change interventions (BCIs) [41]. Here, we review frameworks that categorize digital health technologies based on physical manifestations and functions (both high-level functional roles and specific system features).

#### On the Basis of Physical Manifestations

PT, a technology intentionally designed to change a person’s attitude or behavior, has been categorized into desktop-based, artifact-based, and environment-based systems, based on form factors [42]. Desktop-based systems are those only accessible through traditional personal computers and include Web pages and emails designed for desktop viewing and computer software. Artifact-based systems are usually portable and may include smartphones, wearable devices, and physically embodied agents, such as robot toys. Environment-based systems refer to computing systems built into the physical space or fixed to facilities to capture behaviors of users of the space or facility and to deliver point-of-choice persuasions, such as a system built into a public restroom to detect and encourage handwashing-with-soap behaviors of all toilet users [43].

#### On the Basis of Roles and Functions

The functional triad of PT [39] describes 3 general roles a computer can play in its interaction with the user, namely, a tool that increases user abilities, a medium that delivers content to create experience, and a social actor that evokes social responses especially with animate characteristics.

More recently, detailed system functionalities have been identified that explicitly or implicitly support those roles. For instance, the persuasive system design (PSD) model [38] suggested design principles under the following 4 categories: (1) primary task support, which includes reducing complex behaviors into simpler ones, tunneling experience, tailoring and personalization, self-monitoring, simulation, and rehearsal; (2) dialogue support, including positive reinforcement, reminders, suggestions, similarity, liking, and social role; (3) credibility, including expertise, authority, and trustworthiness; and (4) social support, by mediating social interactions and social influences. Some of these principles correspond to functional roles in the functional triad. For example, the principle of “reductionˮ (ie, reducing complex behavior into simple tasks helps users perform the target behavior) and “self-monitoringˮ (ie, providing means for users to track their performance or status) both enable the system to play the role of a tool. The principle of “simulationˮ (ie, enable users to immediately observe the link between cause and effect) and “social facilitationˮ (ie, providing means for discerning other users who are performing the same behavior) support the role of a medium; the principle of “social roleˮ (ie, adopt a virtual social role) can be directly mapped onto the role of a social actor in the functional triad. It should be noted that although PSD has the merit of supporting requirement engineering, it does not follow a clear hierarchical structure, and the design principles are a combination of behavior change strategies (eg, self-monitoring), functional elements (eg, simulation), and nonfunctional characteristics (eg, similarity and credibility).

Webb and colleagues [41] developed a novel scheme to code modes of delivering internet-based health BCIs into 3 broad categories: (1) automated functions, including the use of an enriched information environment, automated tailored feedback on progress, and automated follow-up reminders and tips; (2) communicative functions, including mediating communication with advisors and peers; and (3) use of supplementary modes. Similar concepts were termed as BIT elements by Mohr and colleagues [40], referring to actual technical instantiations in the intervention that the user interacts with. In addition to those functional components included in Webb’s coding scheme, Mohr and colleagues [40] listed BIT elements appearing in more recent apps such as passive data collection (PDC; ie, data collected with smartphone sensors or external devices or through application programming interfaces [APIs] from other available sources) and logs (ie, data entry field facilitating self-monitoring).

All the abovementioned frameworks will be considered with adaptations wherever necessary in our analysis of the technological aspects of interventions to be reviewed.

## Methods

### Search and Selection

An interdisciplinary literature search was conducted of the following databases: Ovid MEDLINE, Cochrane library, JBI database of systematic reviews, Association for Computing Machinery digital library and Engineering index Compendex. Table 1 lists the databases searched in each field.

Synonyms and subject headings relating to the following terms were applied in various combinations: *office worker, sedentary behavior, technology, workplace* (see Multimedia Appendix 1 for example search strategy). Reference lists of existing reviews [16-22] on workplace SB reduction and PA promotion were hand searched to identify additional eligible studies.

Title, abstracts, and full text of retrieved articles were reviewed for eligibility by applying the following criteria: (1) having office workers in the study sample; (2) targeting SB during work or had proxy measures of workplace SB (objective and/or self-report daily sitting of office workers); (3) involving digital technologies such as mobile and computer apps, digital multimedia contents, wearable activity trackers, and other devices with sensing and computing capabilities in the production, delivery, and/or customization of intervention contents; (4) published in peer-reviewed scientific journals/conference proceedings between 2000 and 2017; and (5) published in the English language.

**Table 1:** Databases searched in each field.

Fields	Databases
Medical and health sciences	Ovid MEDLINE, Cochrane library, and JBI^a^ database of systematic reviews
Computing and engineering	Association for Computing Machinery digital library and Engineering index Compendex
Interdisciplinary	Scopus

^a^JBI: Joanna Briggs Institute.

Observational studies without administering or developing any intervention were excluded, though design research with an explicit intent to inform the development of digital SB interventions was included. Studies were also excluded if digital technologies were only used for purposes other than intervention delivery, such as using digital tools for pre-and poststudy assessments without feeding the data into the intervention content in any way.

### Data Extraction

Full articles of eligible studies were reviewed to extract the following information where possible: publication data (authors, years, countries where the study was conducted, or where the first author was based if the study country was not specified), primary target behavior (SB vs PA vs others), intervention details, study details (eg, study type, participants, data collection methods, and duration), intervention development and research phase, technological features and configurations, and outcomes. Emphasis was placed on 2 types of outcomes pertinent to the design and use of technology: design-related outcomes informative for future iterations of intervention, which typically included satisfaction, usability, technical and process feasibility (eg, reach, dose, and fidelity of delivery), acceptability, engagement, and interactions with the technology, and user-related outcomes such as change in SB, PA, work performance, and perceived enablers for changes.

On the basis of the UK Medical Research Council (MRC) framework for developing and evaluating complex interventions, we categorized the whole article or sections of the articles into respective research phases: development, feasibility and piloting, evaluation, and implementation; we also categorized the intervention based on the phase reported in the latest publication about the intervention (Table 2).

We adapted existing classification frameworks to derive our own coding scheme to annotate the technological aspect of each intervention (Table 3). The framework was primarily based on the BIT model [40], which complemented with elements from other coding schemes/frameworks introduced previously, to cover a broader range of technologies and to reflect the specialty of the workplace setting (eg, the addition of “mediated organizational support and social influencesˮ). Each code in the classification system can be viewed as a distinct technological feature (eg, a data log) implemented to deliver one or more intervention component (eg, self-monitoring of behaviors). A series of codes joined by “and” were used to annotate a technological configuration where several features were integrated to deliver 1 or more intervention component. For instance, an intervention that offered tailored feedback on progress based on users’ self-reported daily step counts was annotated with “DL and ATF.ˮ Notably, “Scheduled promptsˮ (SP) delivered according to real-time user status passively captured by sensing technologies (“PDC and SPˮ) are inherently different from SPs that interrupt users at fixed times throughout the day regardless of the users’ actual sitting time; hence, an additional code of “JITAI” was used to annotate “PDC and SPˮ configurations to highlight the fact that the JITAI approach was present.

**Table 2:** Definitions of the development and research phases.

Phase	Definition and examples
Development phase	Studies could be one of the following: (1) reporting the design and development process of the intervention, following approaches such as Intervention Mapping, participatory design and user-centered design, (2) laboratory studies investigating design-related outcomes (feasibility, usability, and user experience) before the intervention has reached a deployable state of development, and (3) short in-the-wild deployment studies evaluating specific intervention components within a functional prototype before investing in further development.
Feasibility and piloting phase	Studies focused on investigating design-related outcomes of an intervention after it has reached a relatively complete stage of development, where user-related outcomes (behavior change, health and well-being, and productivity) were often measured as secondary outcomes with smaller sample sizes and less rigorous study designs.
Evaluation phase	Studies using a larger sample size and more rigorous study designs to assess important user-related outcomes and establish the efficacy of interventions.
Implementation phase	The intervention has already gone through the evaluation phase and has been used in practice for some time (eg, ≥2 years). As many implementation efforts are not reported, it was expected that this phase would have low representation.

**Table 3:** Links between our codes and categories from existing frameworks.

Codes with descriptions	BIT^a^ elements [40]	Roles in the functional triad [44]	MoD^b^ for internet-based interventions [41]
Digital logs (DL): technology provides a convenient way for the user to enter data, which can be a mobile phone diary for self-monitoring of behaviors or a Web-based questionnaire assessing current behavior and psychological determinants of behaviors.	Log	Tool	N/A^c^
Passive data collection (PDC): use wearable, smartphone-based and environment-based objective monitors to obtain time-stamped SB^e^ records automatically.	Passive data collection	Tool	N/A
Connected devices (CD): one or more external sensing device is connected either wirelessly or with a cable to a central computing device.	N/A	Tool	N/A
Scheduled prompts (SP): break reminders delivered either at fixed intervals or with some schedule adaptive to the real-time user status.	Notification push	Tool, medium and/or social actor	Automated functions: automated follow-up messages (reminders)
Information delivery (ID): one or more forms of digital media with varying richness (text, links, testimonials, videos, or games) is used to present information that is usually static over time (eg, health facts, scripted motivational messages, and practical suggestions).	Information delivery	Medium and/or social actor	Automated functions: use of enriched information environment (eg, links, testimonials, videos, games); Use of supplementary modes (eg, emails and other digital media)
Automated tailored feedback (ATF): feedback on individual behaviors and progress, such as personalized goal setting and recommendations, that usually require some calculations of data input from DL or PDC.	Reports and visualization	Medium and/or social actor	Automated functions*:* automated tailored feedback based on individual progress
Mediated organizational support and social influences (MOSSI): emails conveying managers’ approval, online forums facilitating communication and/or competition among program participants, and other digital means of linking the participant to other individuals for the purpose of social influences or organizational support. (Email access to the support from a consultant or coach should be coded under ID instead)	Messaging	Medium	Communicative functions: access to peer-to-peer support

^a^BIT: behavioral intervention technology.

^b^MOD: mode of delivery.

^c^N/A: not applicable.

^d^BCT: behavior change technique.

^e^SB: sedentary behavior.

### Data Synthesis

Results on study characteristics (ie, publication data, study design, MRC development and research phase, and participants) and intervention characteristics (ie, target behavior, theoretical underpinning, technological design, and MRC development and research phase) were quantitatively summarized and presented using descriptive statistics.

Due to the heterogeneity of study design (eg, interviews, laboratory testing, and randomized controlled trials [RCTs]) and outcomes (eg, design inspirations, usability, engagement, and effectiveness), meta-analysis of specific outcomes across studies was not suitable. Instead, a primarily qualitative approach was used to summarize the research under each category of technological configuration, with a focus on design-related findings and implications, which were most relevant to the research questions of our interest.

## Results

### Overview

**Figure 1:**
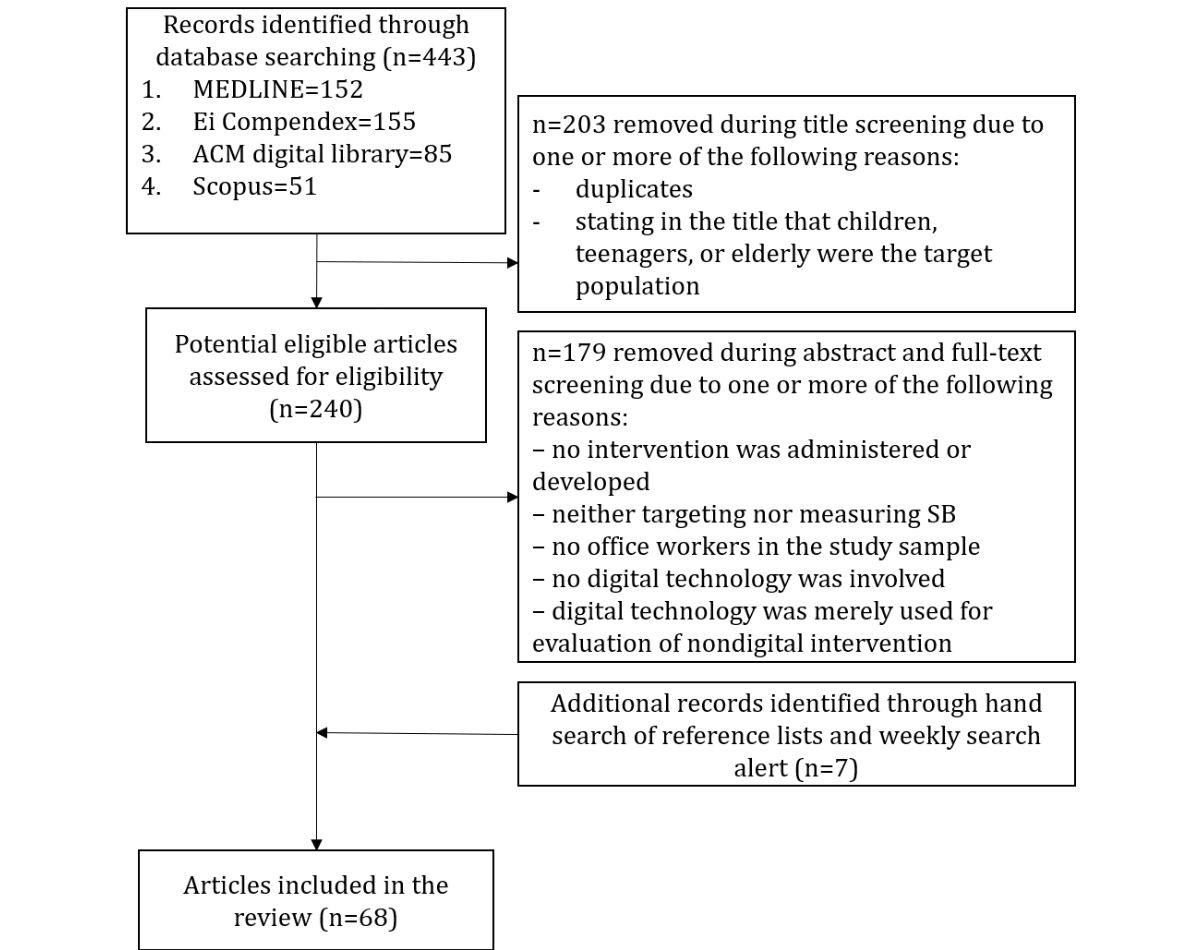
Search and screening results.

A total of 68 articles were included in this review (Figure 1), corresponding to 45 unique interventions. Each article was counted as a separate study, even if it was focused on a different aspect of the same research project reported in another article.

### Study Characteristics

#### Publication Data

**Figure 2:**
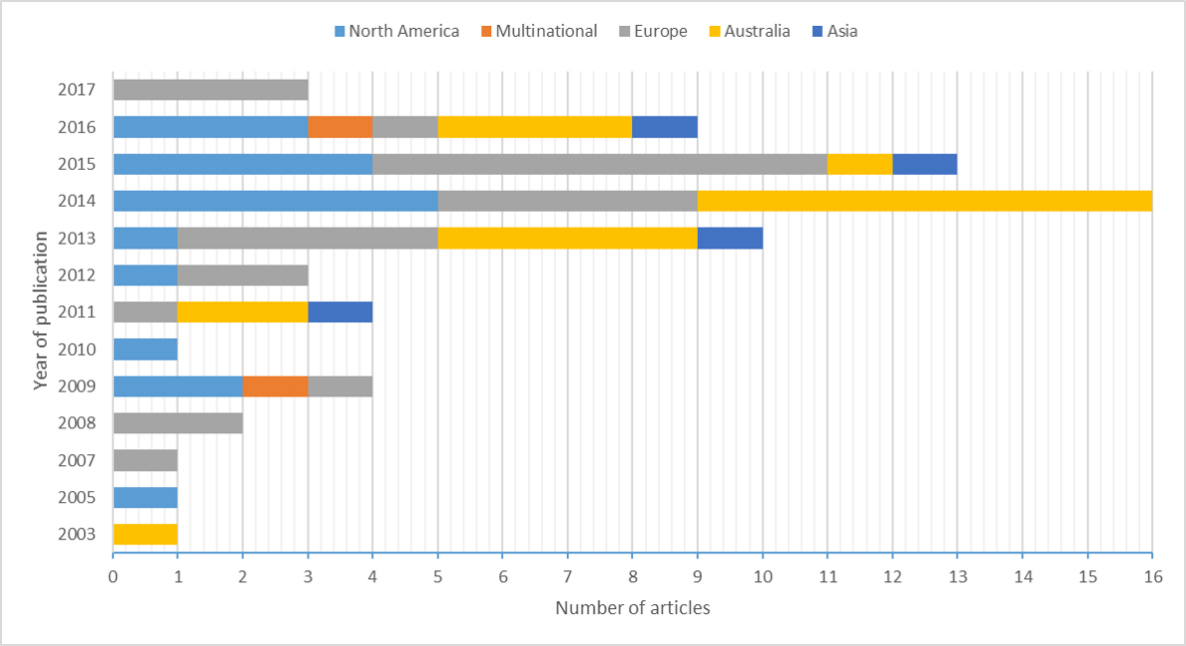
Number of articles by year of publication and country of study.

As shown in Figure 2, there is an overall upward trend in the number of articles published on this topic over the past two decades or so, with 2014 being the most fruitful year. Overall, 66 published articles represented research that was conducted in 16 countries, in addition to 2 articles that reported international studies conducted in 64 countries [45] and 3 countries (the United Kingdom, Australia, and Spain) [23], respectively. The most represented countries were Australia (n=19 articles), the United States (n=17), the Netherlands (n=8), and the United Kingdom (n=4). Another 7 European countries (eg, Austria, Spain, Portugal, Belgium, Germany, Switzerland, and Finland) were represented in a total of 20 articles.

In terms of publication avenues, the included articles were published in 40 different scientific journals and proceedings. Divided by disciplines, 42 articles were published in the field of medical and health sciences, 13 in engineering and computing (including ergonomics and human factors), and 13 in interdisciplinary journals or conferences (eg, *PloS One*), out of which 6 were in the interdisciplinary field of digital health (eg, *Journal of Medical Internet Research*).

#### Study Design

For experimental studies, 25 articles reported RCTs (including cluster RCTs), 4 reported randomized crossover studies, 4 reported before-and-after studies with control or comparison group(s), and 10 reported before-and-after studies without control or comparison group(s). In addition to those traditional experimental designs, 9 articles reported descriptive quantitative process data (eg, fidelity of delivery, reach, usage pattern of the technology, and compliance to break prompts), 11 articles reported qualitative data reflecting participants/stakeholders’ perspectives (eg, pre-and poststudy interviews), and 19 articles reported the design and development of the technology.

Note that the above categories were not mutually exclusive as 1 article could include both quantitative and qualitative results and report both the design process and an evaluation study.

#### Development and Research Phase

All 68 articles featured complex interventions according to the MRC definition. Table 4 shows the number of articles categorized into each intervention development phase based on the MRC framework. Except for 2 articles that reported both the development and piloting phase [46,47], each article was assigned 1 category.

**Table 4:** Distribution of articles by development phases.

Phase of intervention development	Articles (n)
Development	19
Feasibility and piloting	34
Evaluation	10
Implementation	7

#### Participants

All studies included participants employed in office-based jobs. Indeed, most studies recruited participants from office-based workplaces covering different sectors and worksite sizes, although the majority of studies were conducted in universities and public-sector worksites. Only a few design and development studies recruited via local newspaper, social media, and from participant pools, resulting in a mixture of office workers and unemployed participants (eg, the study by Rabbi and colleagues [46]—13 students and 4 office workers; the studies by Bond and colleagues and Thomas and colleagues [48,49]—12 retired/employed and 18 office workers; the study by Mukhtar and Belaid [50]—2 graduate students and 2 faculty members; and the study by He Q and Agu [51]—6 students and 2 colleagues).

Overall, 63 studies recruited participants regardless of body mass index, whereas 5 studies targeted overweight and obese adults [48,49,52-54]; all studies but 1 [55] included both female and male participants. Except for 1 design and development study where sample size was not reported, sample sizes ranged from 1 [56] to 91 [57] among development studies, 3 [55] to 412 [58] among piloting studies, 153 [59] to 631 [60] among evaluation studies, and 291 [61] to 69,291 [45] among implementation studies.

### Intervention Characteristics

#### Target Behavior

Of all 45 interventions, 18 interventions (27 articles) focused primarily on SB reduction, 14 (22 articles) targeted a combination of SB reduction and other behaviors (eg, PA promotion, diet management, posture correction, prompting social interactions with colleagues, and general lifestyle change), and 13 (19 articles) targeted other behaviors (eg, posture correction and PA promotion) without an SB reduction element in the intervention design but reported SB change as a secondary behavioral outcome.

#### Theoretical Underpinning

Overall, 19 interventions were underpinned by at least one theory, which included the theory of planned behavior (n=5), social cognitive theory (n=4), social ecological model (n=4), the stages of change or transtheoretical model (n=4), and theories of habits (n=3). The development of 3 interventions followed frameworks (eg, Intervention Mapping) that supported theory-based intervention design [57,62,63].

#### Technological Design and Development Phase

Multimedia Appendix 2 provides details about the technological features and configurations implemented in each intervention, the methods used to study those interventions, and study outcomes. Table 5 presents summative results on different technological features/configurations in relation to the development and research phase based on MRC framework.

### Summary of Design-Related Findings

#### Information Delivery and Mediated Organizational Support and Social Influences

The use of digital media for “information deliveryˮ was prevalent among reviewed interventions and was sometimes integrated with the feature of “mediated organizational support and social influencesˮ (“ID and MOSSIˮ). A long-standing use case of this was motivational messages sent from the managers’ email addresses to convey organizational support and endorsement for the program [59,64-66]. In other cases, “ID and MOSSIˮ were implemented in the form of online discussion forums or social networking sites to encourage individuals to share experiences with peers and to foster social support or team competition [45,60,61,67,68].

Two-thirds of the “ID and MOSSI” interventions had moved beyond development and piloting phases, with 6 interventions [23,59,60,69-71] having reached the evaluation phase and 2 [45,61] having reached the implementation phase. There was consistent evidence for positive user-related outcomes (eg, reduction in SB and increase in PA and work productivity) across studies [23,59,60,67,70,71], except for the study by van Berkel and colleagues [69], which delivered a lifestyle intervention with a small component focused on SB reduction and yielded nonsignificant intervention effects on SB or other lifestyle behaviors.

The only published development work on “ID and MOSSI” configuration was novel in applying ambient and affective interfaces to persuasion. A system called “PerFrameˮ was created to play footages of the users’ close friend performing expressions showing either approval or disapproval, depending on whether the users’ behavior was healthy or not [72].

#### Digital Log and Automated Tailored Feedback

Integration of “digital logˮ and “automated tailored feedbackˮ was another common configuration (“DL and ATFˮ), as such systems took user inputs and generated feedback accordingly. These ranged from textual advice tailored to psychological constructs assessed with a simple Web-based questionnaire [24,73,74] to sophisticated visualization and simulations tools providing feedback on outcomes of self-reported behaviors such as daily step counts [45,60,67] and PA [75,76].

Although only 8 interventions were identified in this category, half of them [24,60,67,74] had reached the evaluation phase and one [45] the implementation phase. All reported SB reduction in the intervention group over time, though only 2 [60,67] reported significant between-group (intervention vs control) difference in SB reduction.

**Table 5:** Summative results on technological design and development phase.

Technological design	Total, n (%)	Development, n (%)	Feasibility and piloting, n (%)	Evaluation, n (%)	Implementation, n (%)
Overall	45 (100)	13 (29)	21 (47)	8 (18)	3 (7)
ID^a^	36 (100)	9 (25)	17 (47)	8 (22)	2 (6)
DL^b^	14 (100)	1 (7)	5 (36)	5 (36)	3 (21)
PDC^c^	39 (100)	12 (31)	18 (46)	6 (15)	3 (8)
CD^d^	12 (100)	6 (50)	5 (42)	1 (8)	—^e^
SP^f^	28 (100)	13 (46)	14 (50)	1 (4)	—
ATF^g^	29 (100)	9 (31)	12 (41)	6 (21)	2 (7)
MOSSI^h^ and ID	12 (100)	1 (8)	3 (25)	6 (50)	2 (17)
PDC and ATF	26 (100)	9 (35)	11 (42)	4 (15)	2 (8)
PDC and SP (JITAI^i^)	19 (100)	13 (68)	5 (26)	1 (5)	—
Using on-board sensors	8 (100)	6 (75)	2 (59)	—	—
Using connected sensing devices (“CD, PDC, and SP”)	11 (100)	7 (64)	3 (27)	1 (9)	—

^a^ID: information delivery.

^b^DL: digital log.

^c^PDC: passive data collection.

^d^CD: connected device.

^e^no intervention found in the category

^f^SP: scheduled prompts.

^g^ATF: automated tailored feedback.

^h^MOSSI: mediated organizational support and social influences.

^i^JITAI: just-in-time adaptive intervention.

Several studies have examined design-related outcomes such as user engagement and experience of the “DL and ATF” platform. For instance, in the study by Compernolle and colleagues [74], it was reported that 86% of the participants in the intervention condition requested computer-tailored feedback and advice and that the majority rated the advice positively; in contrast, in the study by Marshall and colleagues [24], only half of the participants visited the website for tailored feedback and even fewer used the website for a second time. Although both platforms delivered stage-based advice tailored to participants’ self-reported PA and psychological determinants of PA, it could be the provision of pedometers in the study by Compernolle and colleagues [74] that made a difference.

Despite a lack of evidence showing “DL and ATF” as the efficacious component causing SB reduction, it was reported as a key mechanism of behavior change in several qualitative studies. Participants in the study by Bort-Roig and colleagues [77] highlighted the motivational value of being able to view logged data through visual graphics on a website and gain feedback; the study by Cooley and colleagues [78] interviewed 15 participants, who suggested that the mere act of logging nonpurposeful physical activities during breaks changed their perceptions of what constituted exercise—they also thought the automated feedback on progress helped them set up goals.

#### Passive Data Collection and Automated Tailored Feedback

Replacing “digital logˮ with “passive data collectionˮ to provide input for “automated tailored feedbackˮ is a more technologically advanced configuration (“PDC and ATF”), as it capitalizes on automated sensing technologies and activity detection algorithms. Smartphones and pedometers were the 2 most frequently used devices for this configuration.

A number of smartphone apps incorporated data from on-board accelerometers or utilized Android APIs for real-time activity classification. Feedback was usually offered in the form of a dashboard with a break timer, daily accumulative active and inactive minutes, and/or a lifelog of activity episodes in chronological order [47,48,50,51]. Practical issues with this technological approach were identified, such as “phone battery drained quickly because of the accelerometer useˮ and “users did not always carry the phone with themˮ [47,51,79].

Pedometers were often used to provide instant and simplistic feedback on PA (eg, [74]). They were also used as a support tool (1) alongside DL to enhance the accuracy of self-report PA and (2) alongside MOSSI to provide the metric for team-based competition [28,45,52,54,66,71,80,81]. Participants generally considered the technological monitoring tool very helpful [54,77] and an evidence for organizational investment in staff health [82].

Notably, only 6 [23,45,59,67,74,81] out of the 25 “PDC and ATF” had reached the evaluation and implementation phases, 5 of which were pedometer-based interventions. Most interventions that used smartphone for both “PDC and ATF” were in the development and piloting phase.

Development research conducted in this space was innovative and informative in several aspects. First, machine learning was applied to classify activities and generate suggestions based on the users’ past behavioral patterns, which were found to yield stronger intention to follow than generic suggestions [46]. Second, the likeability of different forms of feedback was explored: “at-a-glanceˮ and real-time display of summative data was perceived as useful and motivating by users [48,51]; potential features demanded by users were visual feedback on the health outcomes of SB, accurate and reliable data sources, and the control over the collection and sharing of their data feedback with colleagues [83].

#### Passive Data Collection and Scheduled Prompts (Just-in-Time Adaptive Interventions)

Passively collected data were utilized in 19 interventions to determine when to trigger prompts. Those were coded as “PDC and SPˮ in addition to “JITAIˮ in a bracket to be differentiated from the 9 SP interventions that prompted users at fixed times throughout the day [52,55,76,84-87]. Smartphone was the top-choice device used in this category, followed by desktop computers. A few studies used other connected devices (CD), which will be discussed in the “CD, PDC, and SP” configuration category.

Overall, 18 out of 19 “PDC and SP” interventions were in the development and piloting phase. This body of research produced outcomes particularly relevant to this review.

First, the studies were fruitful in identifying the optimum modality, frequency, and manner for interrupting users in the middle of sedentary work. Van Dantzig and colleagues [47] suggested the textual content of the persuasive messages was unimportant and a timely tactile notification on the smartphone might be just sufficient. Thomas and Bond [49] conducted a randomized crossover study with audible break prompts delivered from a smartphone app for 1 week in each of the 3 conditions: (1) a 3-min break prompt after 30 continuous sedentary minutes, (2) a 6-min break prompt after 60 sedentary minutes, and (3) a 12-min break prompt after 120 sedentary minutes. It was discovered that the 3- and 6-min conditions resulted in the greatest number and sum duration of walking breaks, the best and fastest compliance with prompts; from the users’ perspective, the 6-min condition was the most preferred one [48]. Mukhtar and Belaid [50] found that reminders delivered with variable intervals adaptive to the duration of the last inactive episode were preferred by users to reminders delivered with fixed intervals. In terms of manner, some interventions adopted a so-called “passive promptˮ approach, in which the screen was locked unless the user complied with the suggestions, whereas others followed an “active promptˮ approach by allowing the user to snooze or dismiss the prompt and carry on work. Although higher odds of compliance were recorded in the passive prompts condition than in the active prompts condition in 1 study [75], user annoyance with the passive prompt approach was also reported [78].

Second, the research was innovative in applying “quick-and-dirtyˮ design methods to piloting novel intervention approaches and studying potential usability issues without large investment in development. For instance, in the abovementioned PerFrame study, a so-called “Wizard of Ozˮ paradigm was applied to control the system output. That is, instead of implementing complex Computer Vision algorithms, the researcher observed the users’ sitting posture via a camera and remotely controlled which video footages to play [72]. In another example, researchers drew on a range of design research techniques such as diary, scenario, and technology probe to elicit user feedback on the design idea of an emotionally expressive robot, which would otherwise take a long period of development before getting users’ input [88].

#### Connected Devices, Passive Data Collection, and Scheduled Prompts

Within the “PDC and SPˮ configuration category, 11 intervention delivery systems employed an even more technologically advanced feature, by drawing on data from externally *CD*.

Only 1 “CD, PDC, and SP” intervention had moved to the evaluation phase [59]. The study compared an intervention including a wearable activity tracker that made the smartphone prompts responsive to real-time user status with an intervention without the external device. Although there were no significant between-condition differences in prolonged sitting reduction, a 70.5% uptake of the waist-worn activity tracker was encouraging.

The development and piloting research in this space extended our knowledge of devices and media that can be possibly used for delivering SB interventions.

Several peripheral sensing devices with various form factors were incorporated in interventions reviewed, including cushions on chairs to monitor sitting time [53,56], wearables to capture activities and postures [59,89,90], and sensors attached to workstations to infer sedentary time from workstation use time [47,91].

A number of data transfer technologies were used to establish connectivity between devices. Bluetooth technology was commonly used for wireless communications between portable devices, for instance, between an Android/iOS device and a nearby peripheral sensing device [59,92]. Some early studies used mobile networks to send text messages from a server to a mobile phone as a way of prompting users [47,89]. Universal Serial Bus (USB) and other cable-based connections were often utilized in systems for which portability was not crucial. For instance, the studies by Van Dantzig and colleagues, Slootmaker and colleagues, Ferreira and colleagues, and Carr and colleagues [47,89,91,93] used USB-type protocols for sending environment-based sensor data to the users’ workstations, where the prompts were scheduled and delivered. USB protocol was also used in early prototypes of connected systems [56,94] to actuate novel user interfaces (eg, mechanically controlled sculpture and ambient light) from an Arduino, which is an open-source platform for creating interactive electronic objects.

Pros and cons of different technologies were explored. Wadhwa and colleagues [79] examined the technological feasibility and social acceptability of mobile versus environment-based sensing. The authors proposed a triggered-sensing approach to replace some mobile sensing with infrastructure sensing to extend battery life of mobile sensors; in addition, they analyzed users’ response latencies to different prompts and found a slight user preference for mobile-based notifications to workstation-based ones. Haller and colleagues [95] connected a posture sensing chair to 3 different types of media for delivering prompts (onscreen graphic feedback, tactile feedback from the chair itself, and physical feedback delivered by a plastic plant that became droopy to represent bad posture of the user); the result was in favor of the physical feedback, as it required the shortest time to return to the main task after the prompted activity and was rated by users as least disturbing. Along the same line of reasoning, several design studies assessed the technological feasibility, ease of understanding, usability, and likeability of ambient displays, such as programmable sculptures that changed shape [56,91], or ambient lights that altered color [94,96] to reflect users’ sedentary time and remind the user to take breaks. Nonetheless, although all the researchers suggested the need for longer-term experiments to establish the viability of their design approaches, no published follow-up studies were found.

## Discussion

### Principal Findings

This review sought to inform its readership about the research activity and technological landscape in the field of digital SB interventions for office workers and to identify research gaps and collaborative opportunities that could be further exploited.

This paper, first of all, serves as a roadmap that indicates the range and location of the literature on this topic. A total of 68 articles describing 45 interventions were identified. Although only a few studies were capable of providing definitive evidence (25 RCTs, of which only 9 were qualified as “evaluationˮ phase studies), this is to be expected in an expanding field of interest with a lot of efforts to bring in novel technological features and configurations. In terms of geographic distribution, we observed that the development and piloting work conducted in this field was located across the globe, whereas evaluation/implementation research tended to be concentrated in specific countries and was usually associated with large national research initiatives (eg, Australia: “Stand up Australiaˮ and “Global Corporate Challengeˮ; the Netherlands: “Vitality in Practiceˮ; and Spain: “Walk@WorkSpainˮ). Some of those projects were also fruitful in generating publications, partly because they followed a phased approach to conducting and reporting the development, piloting, and evaluation of complex interventions as recommended by the MRC guidance (“Stand up Australiaˮ [63,65,70,97,98] and “Vitality in Practice” (VIP) project [57,99]). In terms of disciplines where research on this topic can be located, we demonstrated the added value of searching for articles outside medical and health sciences databases. Finally, we found many SB reduction elements embedded in interventions targeting other behaviors such as posture correction or PA promotion. Indeed, only 18 interventions in this review solely targeted SB reduction.

Second, this review provides an overview of the current technological landscape in this field, with a novel coding scheme constructed specially for this purpose. As shown in Table 5, configurations like “ID and MOSSIˮ and “DL and ATFˮ have mostly been researched in the evaluation and implementation phase. Less investment in development or piloting was observed, probably because those configurations typically used technologies merely as media to exchange information that was traditionally delivered with print media or face-to-face communications, and hence, less complex computational model or infrastructure design was needed. In contrast, research on interventions that delivered SP or ATF based on PDC (“PDC and SP [JITAI],” “PDC and ATF”), in particular with sensors from CDs (“CD, PDC, and SP”), mostly remained in the development and piloting phase.

Notably, although validated PDC devices, such as the ActivPAL (PAL Technologies Ltd, Glasgow, United Kingdom) and ActiGraph (LLC, Pensacola, FL, USA), were widely used for outcome measurement [27,28,52,59,65,70,80,84,87,97,98,100], they were seldom integrated with other technological features as part of the intervention delivery system in the studies reviewed. This might be because early models of the ActivPAL and ActiGraph devices were not equipped with any output module (eg, a screen) to let wearers, or even researchers, receive feedback on SB during the monitoring period; neither were the stored data accessible to third-party apps or devices in real time for implementation of JITAI. This may, in turn, demotivate deployment of those devices beyond the assessment period (usually 1 week or 5 workdays), which could otherwise collect data throughout the whole study period and generate valuable insights into the process of change, as demonstrated in several studies [47,49,97]. This situation should soon be improved with the latest ActiGraph GT9X Link (LLC, Pensacola, FL, USA) and SitFIT (PAL Technologies Ltd, Glasgow, United Kingdom) devices that come with screens for instant feedback on behaviors and Bluetooth modules for communication with external devices.

That was why we coded CDs separately and considered it a very important trend that could potentially catalyze a paradigm shift in the use of data in behavior change. Not to mention easier integration of multiple data sources to make interventions more relevant to the context, CD greatly expands the range of interfaces and media that can be used to deliver SPs to users. We identified exploratory work on developing and piloting ambient displays to deliver break reminders subtly [72,94,96]. The technological advancements in the field of Tangible, Embedded, and Embodied Interactions presents new promise for this line of research, as mechanically controlled objects have been created [56,95] or designed [88,91] as a creative and pleasant way to persuade users into taking breaks and caring for their own health.

### Implications

In addition, 2 notable blank spots can be identified in Table 5, suggesting areas where evidence is lacking and more investigations are warranted.

One is the dearth of research on interventions utilizing CDs, especially in evaluation and implementation phases. Research opportunities exist in exploiting wireless connectivity to make interventions more relevant to individual users and contexts. Manufacturers of well-validated PDC devices are starting to provide Software Development Kits (SDKs), such as the new ActiGraph Link SDK, which allows third-party apps or devices to stream the PDC devices’ raw data in real time or near real time. This is very encouraging; however, no studies have been published featuring interventions using such SDKs to exploit the value of CD. To achieve this, collaborations between health scientists, computer scientists, and engineers from both academia and the industry need to be fostered.

Another notable blank spot in Table 5 is the lack of research on SPs beyond the piloting/feasibility phase. Considering the numerous innovative break-prompting installations that have been developed and piloted in engineering and computer science, efforts could be directed toward moving them to the next phase of evaluation with a more rigorous study design. This line of research is promising for 2 reasons. First, research suggests in-the-moment guidance that prompts smaller yet more frequent changes in existing behavior has a potential for greater impact than suggestions only tailored to overall behaviors periodically (eg, daily energy burnt) [46]. However, there is a lack of knowledge about the opportune manner of prompting office workers in the moment of sedentary work. Second, as the cost of embedded electronics is dropping, it becomes increasingly possible to scale up interventions delivered with novel technological devices, such as those systems reminding users subtly by changing ambient light or shape of physical artifacts [56,94,96].

Finally, upon reflection of using the MRC framework and conducting this review as an interdisciplinary team, we have realized differences in the understanding of “developmentˮ and a lack of connection between different communities. There are encouraging examples where researchers followed through and published more than 1 stage of developing, piloting, and evaluating an intervention [57,62,63]. However, when it comes to the design and development of technologies for delivering interventions, it appears that health and behavioral scientists without technical backgrounds are less involved or interested. Meanwhile, although technological innovations are taking place in the fields of engineering and design, there seems to be a lack of mechanisms in place to feed design-related findings into other fields or move the novel technologies downstream to the evaluation phase.

It requires more thinking as to how to better connect and empower 2 communities—the community with expertise in intervention content development and evaluation and the community with capacities to design, develop, and study technologies with users. The answer to the question is beyond the scope of this review. Nevertheless, as a starting point, researchers from all disciplines can familiarize themselves with the MRC guidance and position their research in the big picture of developing and evaluating complex interventions. Health and behavioral scientists can also get more involved in user-centered research and have more inputs to early-stage technology innovations.

### Limitations

The aim of this review was to scope the research activities and describe the technology design in SB interventions targeting office workers; as such, we did not intend to compare or synthesize the behavior change outcomes across interventions with meta-analysis. In addition, our review used a single code for PDC and focused on its integration with other technological features. The measurement and self-monitoring properties of different devices used in those studies could have been coded with a more fine-grained coding scheme. However, we deemed this unnecessary, because a scoping review specifically on devices for self-monitoring SB and PA [36] was published during our data extraction phase and the authors of that review had coded the devices in terms of wear locations, outcomes measured, the type of feedback available, and various other measurement and self-monitoring properties

### Conclusions

This review demonstrates the prevalent and diverse use of digital technologies in SB interventions targeting office workers. The use of technology to deliver information, to mediate organizational support and social influences, and to provide feedback based on self-reported data is well established in this field. More research is needed to exploit wireless connectivity between devices to make interventions more adaptive to the users’ current state and context. Novel media interfaces for delivering subtle prompts are being innovated and are worth more attention. Opportunities exist to improve the utility of future research by encouraging interdisciplinary conversations and collaborations, potentially under the MRC framework for the development and evaluation of complex interventions.

## Appendix

Multimedia Appendix 1 Search strategies.

Multimedia Appendix 2 Summary of technological design, study method and key results.

## Abbreviations

API: application programming interface
ATF: automated tailored feedback
BCI: behavior change intervention
BCT: behavior change technique
BIT: behavioral intervention technology
CD: connected device
DL: digital log
ICT: information and communication technology
ID: information delivery
JBI: Joanna Briggs Institute
JITAI: just-in-time adaptive intervention
MoD: mode of delivery
MOSSI: mediated organizational support and social influences
MRC: Medical Research Council
MVPA: moderate to vigorous physical activity
PA: physical activity
PDC: passive data collection
PSD: persuasive system design
PT: persuasive technology
RCT: randomized controlled trial
SB: sedentary behavior
SDK: Software Development Kits
SP: scheduled prompts
USB: Universal Serial Bus
