# Supplementary material for: Digital Interventions to Reduce Sedentary Behaviors of Office Workers: Scoping Review
Source: J Med Internet Res. 2019 Feb 7;21(2):e11079. doi: 10.2196/11079 (PMC6383116; doi:10.2196/11079)
Supplement: Multimedia Appendix 1 [file jmir_v21i2e11079_app1.pdf]

MEDLINE Search Strategy

#	search term	fields searched	notes for each search	hits
1	exp Internet/ or exp Computers, Handheld/ or exp Telemedicine/ or exp Cell Phones/	subject headings	different digital media	93,517
2	exp Monitoring, Physiologic/ or exp Monitoring, Ambulatory/ or exp accelerometry	subject headings	monitoring techniques	158,104
3	(((mobile or smart or cell) adj2 (app or phone* or telephone* or technolog*)) or pedometer* or accelerometer* or Bluetooth or wireless or ((digital or connected) adj2 (health)) or (mhealth) or (ehealth) or ((information* or digital) adj3 (technolog* or media))or (software) or ((computer* or screen?) adj2 prompt*) or(email*) or(website*)).mp.	keyword	different digital media, devices and applications	55,007
4	#1 or #2 or #3		**involving digital tech**	284,380
5	(sedentary or sitting or screen time or computer time).mp.	keyword	**involving SB or related behaviours**	44,890
6	#4 and #5		tech for SB	3,811
7	("office work*" or white-collar or worksite* or workplace* or occupational health).mp.	keyword	**focused on office workers or workplace context**	46,988
8	#6 and #7		tech used on office workers for SB-related purposes	140
9	(jitai or "just in time adaptive intervention").mp.	keyword		18
10	#8 or #9		include JITAI	156
11	limit 10 to (yr="2000 - 2017" and english)		published in 2000 - 2017, and in English	152

Ei Compendex Search Strategy

#	search term	fields searched	notes for each search	hits
1	(($digital $media OR $Internet OR $web OR $smartphone OR $wearable OR acceleromet* OR $pedometer OR Bluetooth OR $wireless OR $intelligent OR $sensor OR $sensing OR $computing OR {m-health} OR {e-health} OR $software OR {internet of thing} OR {IoT}) WN KY)	title/abstract/key	**involving digital tech**	5,761,508
2	(((reduce OR increase OR decrease OR improve OR change OR modify OR modify OR encourage OR discourage OR motivate OR persuade OR prompt OR remind OR mitigate OR manipulate) AND ($sedentary OR $inactivity OR $sitting OR {computer work} OR {office work} OR {desk work} OR $break)) WN KY)	title/abstract/key	**changing SB or related behaviours**	47,677
3	({office worker} OR {working age} OR workplace* OR worksite* OR employee* OR adult* NOT teenag* NOT child* NOT $elderly NOT adolescen* NOT student*)WN KY	title/abstract/key	**involving office workers or related populations**	86,750
4	#1 AND #2 AND # 3		tech for changing SB or related behaviours in office workers or related populations	164

5	#4 NOT ((1998 OR 1997 OR 1995 OR 1993 OR 1992 OR 1986 OR 1976) WN YR) AND({english} WN LA)	years and langua	published in 2000 - 2017, and in English	155

Scopus search strategy

#	search term	fields searched	notes for each search	hits
1	TITLE-ABS-KEY (("digital medi*" OR internet OR web OR smartphone OR wearable OR acceleromet* OR pedometer OR Bluetooth OR wireless OR intelligent OR sensor OR sensing OR computing OR "m-health" OR "e-health" OR software OR "internet of thing" OR "IoT"))	title/abstract/keyword	**involving digital tech**	4,054,597
2	TITLE-ABS-KEY((reduce OR increase OR decrease OR improve OR change OR modify OR modify OR encourage OR discourage OR motivate OR persuade OR prompt OR remind OR mitigate OR manipulate OR "break up") W/1 (sedentary OR inactivity OR sitting OR "computer work" OR "office work" OR "desk work"))	title/abstract/keyword	**changing SB or related behaviours**	1,768
3	TITLE-ABS-KEY("office work*" OR "working age" OR workplace* OR worksite* OR employee* OR adult* AND NOT teenag* AND NOT child* AND NOT adolescen* AND NOT student*)		**involving office workers or related populations**	5,629,832
4	#1 AND #2 AND #3		tech for changing SB or related behaviours in office workers or related populations	153
5	#4 AND NOT INDEX(medline)		excluding MEDLINE indexed	53

6	#5 AND (LIMIT-TO (LANGUAGE , "English")) AND PUBYEAR > 1999 AND PUBYEAR < 2018		published in 2000 - 2017, and in English	51

ACM Digital Library search terms

#	search term	fields searched	notes for each search	hits
1	sedentary sitting break) AND acmdlTitle:(combat persuade remind change reduce increase motivate intervention)	full-text and abstr	simple search of articles on changing SB or related behaviours	102
2	filter: {"publicationYear":{ "gte":2000, "lte":2017}}		published in 2000 - 2017	85

Note on red coloured search terms: In the medical/health sciences literature, we were particularly interested in the appropriateness and acceptability of different digital SB interventions in office-based workplaces. So we limited the MEDLINE search to ‘workplaces’ and ‘office work’, and it served our needs to pick up relevant studies, while leaving out those without an occupational focus.
As for the engineering computer science literature, we were more interested in design-related findings (e.g. usability, UX and technical feasibility) about SB interventions or SB reduction component in interventions intended for other purposes (e.g. screen break, lifestyle change, weight loss) we decided to expand the Compendex/Scopus/ACM search to include SB-related interventions not explicitly targeting office workers or workplace SB and made sure only interventions with components that actually addressed SB at work and tested with a sample that included at least 1 office worker were included during manual screening.
