# Supplementary material for: Digital Interventions to Reduce Sedentary Behaviors of Office Workers: Scoping Review
Source: J Med Internet Res. 2019 Feb 7;21(2):e11079. doi: 10.2196/11079 (PMC6383116; doi:10.2196/11079)
Supplement: Multimedia Appendix 2 [file jmir_v21i2e11079_app2.pdf]

Multimedia Appendix 2. Summary of technological design, study method and key results

Study	Coded technological features and configurations	Study type and design	Results
(Priebe and Spink, 2015)	ID & MOSSI: email messages containing descriptive norms about co-workers sedentary and light physical activity	Pre-post with comparison groups (2 (personal similarity: high vs. low) ×2(contextual similarity: high vs. low)) n=142	**User-related:** Lower self-report sedentary behaviour, greater stair use and walking were observed in those who received descriptive norm messages about co-workers' healthy office behaviours. No differences between groups.
(Neuhaus, Genevieve N Healy, *et al.*, 2014)	ID & MOSSI: manager's emails promoting the study, noting the progress and providing tips; individual emails summarising goals<br>PDC: activPAL for outcome measurement<br>(for 1 week, baseline and 4-week follow up)	Design & development	**Design-related:** Improvement to each element after formative and piloting phase was reported
(Healy *et al.*, 2013)		Pre-post with control (cluster)<br>(I=19; C=19)	**User-related:** activPAL captured sig. reduction (in I group relative to C) in sitting (-125 min/workday,) and sitting > 30 min (−74 min /workday) and 2 more sit–stand transitions per hour
(Stephens *et al.*, 2014)		Descriptive quantitative (Timestamped activPAL data was analysed to see how and when changes occur)	**User-related:** Sig. reduction (I relative to C) at all hours of the workday, except for 12:00- 13:00. The large differences (≥30% reduction in workplace time spent sitting) were evident before 9:00, 9:00-10:00 and 11:00-12:00
(Neuhaus, Genevieve N. Healy, *et al.*, 2014)	Same as above, except for follow-up schedule:<br>(for 1 week: baseline and 3-month follow-up)	RCT (cluster, quasi-randomised)<br>(I_1(multi-component) =16; I_2(SSD-only) =14; C =14)	**User-related:** Sig. reduction in sitting (-89 min/workday, -56min/workday) in I_1 compared to C and I_2; I_1 group rated additional components and manager emails as useful to very useful.<br>**Design-related:** All I_1 group participants rated additional intervention components as either useful or very useful, and 12/13 rated the manager emails as either useful or very useful
(Healy *et al.*, 2016)	Same as above except for measurement period:<br>(For 1 week, baseline, 3-month and 12-month follow up)	RCT (cluster)<br>(n=231, I=7 worksites, C=7 worksites)	**User-related: :** Sig. reduction in sitting at 3-month (-99.1 min/ workday) and 12-month (-45.4 min/ workday) follow-up

(Brakenridge *et al.*, 2016)	PDC & CD & SP (JITAI): prompts on sitting and posture based on LUMOback belt data connected to smartphone<br>PDC & CD & ATF: LUMOback belt with connected smartphone App for summary on workplace sitting, standing, stepping, breaks;<br>ID & MOSSI: emails delivering information booklet ('Stand Up Lendlease') and feedback on baseline assessment; communicating senior executives' participation and support to employees.<br>PDC: activPAL for outcome measurement (1 week baseline, 3-month and 12-month follow-up)	RCT (cluster) (I (with activity tracker) = 66 (9 teams) C (organisational support only), = 87 (9 teams)	**Design-related:** 70.5% uptake of LUMOback in those provided with the device; variable usage in the first 12 weeks.<br>**User-related:** The following changes were observed within both groups, but only at 12 months: within-work sitting (I: - 35.5 min/workday; C: -40.5 min/workday), prolonged sitting (I: -45.7 min/workday; C: -41.3 min/workday), within-work standing (I: +27.4min/workday; C: +39.2 min/workday). There was no sig. difference between groups on the above. Observed changes in health and work outcomes were small and n.s..
(van Berkel *et al.*, 2011)	'Mindful Vitality in Practice (VIP)'<br>PDC: Actigraph accelerometer for outcome measurement (baseline week, 6-month and 12-month follow-up)<br>MOSSI: e-coaching focused on lifestyle change and mindfulness with certified trainer via Emails<br>ID: email containing information on lunch walking routes.	Design & development (interview (n=6), survey (n=78), focus groups (n=39; 6 groups)	**Design-related:** determinants of PA in leisure time and for SB at work identified as follows: perceived behavioural control, perceived barriers (especially lack of time), and social support; For fruit and vegetable intake, the most important determinants were: habit, perceived behavioural control, availability, cost, and intention.
(van Berkel *et al.*, 2014)		RCT (I = 129, C = 128)	**User-related:** No sig. effect of the intervention on SB, other lifestyle behaviours and behavioural determinants after 6 and 12 months.
(Coffeng *et al.*, 2012)	Be Active & Relax Programme, part of the "Vitality in Practice" (VIP) project, with intervention components similar to van Berker and colleagues'.	Design & development (interview (n=6), survey (n =91), focus group (n=28; 5 groups)	**Design-related:** Determinants of PA identified as follows: attitude, subjective norm, perceived behavioural control, perceived barriers and physical environment; The determinants for relaxation were: awareness, attitude, subjective norm, perceived behavioural control and physical environment.
(Coffeng *et al.*, 2014)		RCT (2 ×2 factorial design, n=412)	**User-related:** Sig. reduction in SB at work were found in the following conditions relative to control: combined intervention (n=92), social intervention (n=118), and physical intervention (n=96); sig. increase in small breaks were found in combined intervention and social intervention groups relative to control.
(Coffeng *et al.*, 2013)		Descriptive quantitative/ process evaluation (monitoring data + survey; n=306, 3 interventions groups)	**Design-related:** the component of step counter reached 50% - 100% of participants and was rated as satisfactory, but were used infrequently; the social media platform component reached 0% -34% of participants, with low satisfaction rating and self-report frequency of use.

(Marshall *et al.*, 2003)	ID & DL & ATF: : fortnightly motivational emails with links to an interactive and animated website with password access and quizzes, feedback on responses, personalised tips on BC strategies over 8 weeks	RCT (C (print)=262, I (web)=250)	**User-related:** IPAQ captured sig. sitting reduction from baseline in Web group (-21 min/workday); **Design-related: Engagement-**half in web group did not view the website at all; lower chance of retaining the material for future reference than print group; a sequential decline of website use and less than 1/2 click-through the link in emails; only 26% log more than once into the website, which were different from previous studies with volunteer participants; **usability -** 9 reported usability issues ; majority were confident about using computer; **preference -** receiving health-related information via email and website is preferred to printed version
(Gilson *et al.*, 2007)	ID & MOSSI: weekly emails containing motivational messages (C), suggested campus walks and maps (route group), tips on increasing steps in the office and in work-tasks (incidental group)<br>PDC & ATF: Yamax SW200 pedometer for real-time feedback on step counts<br>(baseline week and 10-week intervention period)	RCT (waitlist control) (C = 22 ; I_1 (route-based walking group) = 21, I_2	**User-related:** sig. increase in daily step in I_1 and I_2, decrease in C.
(Gilson, McKenna and Cooke, 2008)		Qualitative (interview with 15 participants (7 from I_1, 8 from I_2)	**Design-related:** employees perceived the intervention as an institutional investment in staff; pedometers and weekly e-mail motivational messages provided tangible evidence of the level of investment; **User-related:** also heightened self-awareness of personal health, well-being, and physical activity.
(Gilson *et al.*, 2009)		RCT (waitlist control) (C= 60 ; I_1 (route-based walking group) = 60, I_2 (incidental walking group) = 59)	**User-related:** n.s. group by time interaction for daily recall of sitting time recorded through logbooks at baseline, 1-week, 5-week and 10-week follow-up; sig. increase in daily step in I_1 and I_2, n.s. change in C.
(Parry *et al.*, 2013)	ID: tailored emails with tips on increasing steps in the office and in work-tasks (I_1) and encouraging PA breaks (I_2)<br>PDC: ActiGraph GT3x for outcome measurement (1 week at baseline and 12-week follow-up (end of intervention))<br>PDC & ATF: Yamax Digi-walker SW700 pedometer for real-time feedback on step counts as a motivational tool (I_2 only)	Cluster stratified RCT (I_1 (active workstation) = 19; I_2 (pedometer challenge) = 14; I_3 (ergonomic training) = 29))	**User-related:** A sig. reduction in the percentage of SB on during work hours (-1.7%, -8 min) with n.s. difference across 3 interventions (I_1 (-3.1%), I_2(-0.6%), I_3(-1.4%)) **Design-related:** Least change in organisations involving call centre and data processing work; feedback indicated interventions were not fully supported by management; call for change in organisational culture.

(De Cocker *et al.*, 2015)	ID & & DL & ATF: Email with link to a website with personal log-in and password, where psychological determinants of SB were assessed with a web-based questionnaire; then advice on reducing workplace sitting divided into sections targeting different aspects of workplace sitting and supporting action planning, tailored to current SB was presented.	Descriptive quantitative (monitoring data + survey) (n=179 contacted; n=47 responded to evaluation survey)	**Design-related: Feasibility and reach -** 90 employees requested the advice website; high education, part-time employment, less than 2 standing breaks/hour, positive attitudes positively predicted likelihood to request the advice. **Acceptability -** The majority found the advice interesting, relevant and motivating. Less than one third believed the advice was practicable. After completing the advice, 58.0 % reported to have started interrupting their sitting and 32.6 % additionally intended to do so; 14.0 % reported to have reduced their sitting and another 51.2 % intended to do so.
(De Cocker *et al.*, 2016)	The same ID & DL & ATF as above for I_1<br>ID for I_2: automated generic tips on reducing sitting<br>PDC: ActivPAL for outcome measurement (1 week at baseline, 1-month and 3-month follow-up)	RCT (cluster) (I_1(automated tailored-feedback) = 36; I_2 (automated generic feedback) = 64; C (waitlist control) = 28)	**User-related:** Sig. reduction in self-report sitting in I_1 at 1-month (-12min/workday) and 3-month (-102 min/workday) follow-up relative to control group. Borderline sig. reduction in self-report sitting in I_2 relative to control group. ActivPAL did not capture sig. between-group difference in changes in sitting.
(Compernolle *et al.*, 2015)	PDC: blinded pedometer for outcome measurement, 1 week at baseline, 1-month and 3-month follow-up;<br>PDC & ATF: non-blinded pedometer for feedback, 3 months<br>ID & DL & ATF: Email with link to a website with personal log-in and password, where psychological determinants of PA were assessed with a web-based questionnaire; website delivered advice for reaching 10K steps/day, tailored to psychosocial determinants and average daily step counts during baseline week calculated by the researcher	RCT (cluster) (I = 91; C (not receiving any component) = 107)	**Design-related:** 86% requested the tailored feedback; majority rated the advice as interesting, credible, understandable and instructive but also too long.<br>**User-related:** n.s. intervention effect for self-reported SB and PA
(Kerr *et al.*, 2016)	PDC: activPAL for outcome measurement (SB and sit-to-stand transitions), with which the researcher produced graphical feedback, which was reviewed together with the participant in weekly meetings<br>SP (optional tools): timers (phone apps, computer apps), vibrating watch, emails/texts/phone calls from researcher.<br>DL (optional tools): electronic counters to help self-track sit-to-stand transitions	Mixed-methods<br><br>Two-arm randomised trials (I_1 (decrease sitting) = 15; I_2 (increase sit-to-stand transitions) =15); + Interview	**User-related:** I_1 sig. reduced their daily sitting (-130 min/day), I_2 had no change in sitting time ($p < .001$). I_2 increased their sit-to-stand transitions (+13 transitions/day), I_1 did not.<br>**Design-related:** Recruitment, assessments, and intervention delivery were feasible. Graphical feedback and regular cues to behaviour change (e.g. phone alert) was found helpful; remembering to set reminder and integrating the targeted behaviour change into activities that were outside their daily routines was challenging.

(Aittasalo *et al.*, 2017)	PDC: hip-worn Hookie accelerometer for outcome measurement 1 week at baseline and after the 12-month programme; with which the researcher produced graphical feedback on PA and SB, to motivate participants' wearing and to facilitate goal setting and action planning<br>DL: choice to use a website to monitor their PA<br>MOSSI: choice to use a website to share information with their workmates and friends.	Pre-post (n=296, 12 workplaces)	**Design-related: Fidelity of delivery -** Multilevel implementation at all three levels was applied in six workplaces, while five workplaces implemented actions at two levels and one workplace at one level.<br>**User-related:** Self-report workplace SB decreased from baseline to follow-up (-30min/workday; -22%). Accelerometer measured workplace SB also decreased (-44.9 min/workday;-7.6%). Number of levels or actions had no effect on changes.
(Ganesan *et al.*, 2016)	Stepathlon Programe<br>PDC & DL & ATF: Pedometer for tracking and feedback on step counts; website/mobile App where participants entered daily activity and received interactive advice<br>ID: Daily encouragement e-mails about physical activity and nutrition.<br>MOSSI: online community of participants, with team-based competition and user-generated comments on participation.	Pre-post (n=69219)	**User-related**: Improvements in self-report step count, exercise days, sitting duration (-44,4 min/day), and weight.
(R. LA Freak-Poli *et al.*, 2011)	Global Corporate Challenge<br>ID: motivational emails encouraging increasing step count<br>DL: record step counts via a website or a smartphone application<br>PDC & ATF: pedometer provided outcome measurement and feedback on step counts (throughout the 4-month intervention)	Descriptive quantitative (n=671)	**Design-related:** Reach: employees who started with better health, potentially due to lifestyle or recent behavioural changes, were more likely to respond positively to the program.
(R. Freak-Poli *et al.*, 2011)		Pre-post (n=491)	**User-related:** Sig. reduction from baseline in sitting time (−36 min/workday) immediately after the 4-month programme
(Freak-Poli *et al.*, 2013)		Pre-post (n=315)	**User-related:** Sustained improvements 8-month postprogramme were observed for self-reported sitting time and independently measured blood pressure.
(Freak-Poli *et al.*, 2014)		Pre-post (n=407)	**User-related:** Clinically relevant immediate and sustained (8-month) improvements in wellbeing were observed after participation in the health program.
(MacNiven *et al.*, 2015)		Pre-post (n=587)	**User-related:** Self-report daily sitting time sig. reduced (-21 min/day) from baseline at 16-week follow-up. Pedometer step counts increased sig. between month 1 and month 4, with higher odds of increase in those with low baseline sitting time at work.

(Sternfeld *et al.*, 2009)	ID & MOSSI: 16-week email-delivered programme that allows the user to choose from 4 to 6 tailored small step goals, which will lead to a personal homepage with tips for achieving the selected goals along with modules; discussion board<br>DL & ATF: A progress tracking tool and simulation tool that gives feedback on the outcome of particular behaviour changes in relation to national recommendations	RCT (cluster) (I (physical activity path) = 195; C=436)	**User-related:** The intervention group (in the PA path) self-reported a decrease in sedentary behaviour immediate post-intervention (-11.3 min/day) and 4-month post-intervention (larger effect than (-11.3/min), but data not shown) relative to control group, based on intent-to-treat analysis. A larger change was observed in those who did not meet PA recommendations at baseline.
(Puig-Ribera *et al.*, 2015)	ID & MOSSI: web pages and emails deliver health facts, motivational messages ('sitting less and moving more') and practical tips on increasing incidental movements and short walks; website provided social networking for sharing experiences<br>PDC & ATF: Yamax SW-200 pedometer for step counts (5 workings days at each of the following stages: baseline (1 week), ramping phase (8 week), maintenance phase (11 weeks) and 2-month post-intervention (2 weeks))<br>DL & ATF: website allows the employee to log daily step counts into a personal account; graphic feedback on progress	RCT (C (maintain habitual behaviour with access to pedometer, paper diary and self-reporting sitting time) = 135, I=129)	**User-related:** I group decreased self-report daily occupational sitting at end of ramping phase (-20.6 min/day), and maintenance phase (- 23.5 min/day) and at follow-up (-32.2 min/day), increased step counts and decreased waist circumference from baseline; although sig. between-group difference for sitting time (-22 min) only existed at the end of maintenance phase. Step increase was associated with reduced waist circumference.
(Bort-Roig *et al.*, 2014)		Mixed method (n=12 for interview; n=88 for survey)	**Design-related:** 'active work tasks' and 'increases in walking intensity' were the most frequently used strategies; 'walk-talk meetings' and 'lunchtime walking groups' were the least used strategies; 'sitting time and step count logging' was the most important enabler of behaviour change (highlight the motivational value of being able to view logged data (DL) through visual graphics in a website and gain feedback (ATF); 'screen-based work', 'inherent time pressures' and 'cultural norms dictating sedentary work practices' identified as main barriers.
(Puig-Ribera *et al.*, 2017)		RCT (C (maintain habitual behaviour with access to pedometer, paper diary and self-reporting sitting time = 135, I=129)	**User-related: efficiency-related outcomes -** the W@WS intervention attenuated presentism and loss of work productivity in I group across time relative to C group, while having no impact on mental wellbeing. Better performance was linked to employees being more active, and younger, with higher total sitting time during nonworking days and lower sitting time during workdays.

(Rabbi *et al.*, 2015)	PDC & DL & ATF: feature extracted from Android smartphone accelerometer and GPS sensor data, clustered into four physical activities based on a machine-learning model—Gaussian Mixture Model (GMM); manually logging physical activities, location and food; real-time feedback on behaviour in the form of a life log (a chronological list of events), including feedback on duration of sedentary and active bouts; prioritize goals based on users' past physical activities and food intake, which include suggestions for reducing SB (e.g. 'take a 3 min break each hour').	Design & development; Mixed Methods (RCT (I (tailored) = 9; C(generic) = 8) + Web-based daily diary + interview + survey)	**User-related:** I group walked significantly more than C over the 3 weeks of the study.<br>**Design-related:** Survey showed personalized suggestions yielded stronger intention to follow than generic suggestions. Interview data suggested users in different stages of change benefited differently. Contemplators: considered MyBehavior suggestions as actionable and relevant to their lives, made then more self-conscious about behaviours; Maintainers: considered the feedback as reinforcement and made them want to change SB in office
(Slootmaker *et al.*, 2009)	PDC & ATF: accelerometer-based Physical Activity Monitor (PAM model AM101, PAM BV, Doorwerth, the Netherlands) is) displayed a cumulative score of PA in real time<br>PDC & CD & ATF: PA score could be transmitted to the software and PAM COACH database via a docking station connected to a PC; PAM COACH website could provide more detailed feedback on progress and tailored PA advice	RCT (I (PAM & Coach) = 51; C (information brochure with PA recommendation=51)	**User-related:** n.s. intervention effect was observed for self-report SB and PA.<br>**Design-related:** satisfactory level of use frequency of the website; only 52% set personal goals; more attention should be given to the quality and appropriateness of tailored advice.
(J. Carr *et al.*, 2014)	PDC & CD & ATF: A seated active workstation with built-in sensing of pedalling activities connected to a desk-mountable display monitor that provides real-time biofeedback on pedalling distance, calories burned and cadence and allows for adjustment of resistance in the range of 1-100Watts.	Design & development (lab study + survey)<br>Phase1: n= 45;<br>Phase 2: n=17	**Design-related:** 96% of participants reported they would use the seated active workstation "daily" if provided access in their office; working while using the seated active workstation increased energy expenditure and had no adverse impact on cognitive performance or typing performance, but it did impair mouse clicking ability.
(Carr, Walaska and Marcus, 2012)	PDC & CD & ATF: In 4-week intervention period, a pedalling device (MagneTrainer mini exercise bike) that could be placed under the desk and provide objective measure of pedalling behaviours (time, distance, calorie, speed); data could be transmitted to PC software which stored and provided feedback on daily performance.	Descriptive quantitative (n=18)	**User-related:** No sig. difference in self-reported SB and PA from baseline. no negative impact on work productivity and quality of work was reported.<br>**Design-related**: High acceptability; Participants used the pedal machines 12.2 days out of 20 possible working days and pedalled an average of 23.4 min/day and consumed 186.5 Kcal/day on days they used the machine.
(Carr *et al.*, 2013)	PDC & CD & ATF: same as above but for 12 weeks<br>PDC & ATF: Omron HJ-150 pedometer for feedback on step counts used in conjunction with the website<br>ID & DL: email reminders to use a motivational website (Walker Tracker) where users logged step counts and receive generic tips and reminders focused on reducing sedentary behaviours throughout the day<br>ID & MOSSI: Online forum for posting profile photos and status updates and sending messages to members of their small groups.<br>PDC: Orthocare ankle-worn StepWatch for outcome measurement (0 steps/min as SB) for 1 week at baseline and 12-week follow up.	RCT (I = 23; C (waitlist) = 17) & descriptive quantitative	**User-related:** I group reduced SB (−58.7 min/day) and reduced waist circumference (-2 cm) relative to C post-intervention, after adjusting for baseline values and monitor wear time.<br>**Design-related:** I participants logged on to the website 71.3% of all intervention days, used the pedal machine 37.7% of all working intervention days and pedalled an average of 31.1 min/day; the pedal machine biofeedback display, the pedometer (ATF) and self-monitoring activity on the website (DL) rated as 'extremely helpful'; the email reminders to log daily activity (ID) and access to the pedal machine rated as 'quite helpful'.

(Carr *et al.*, 2015)	PDC & CD & ATF (16 weeks): portable seated elliptical machine (activeLife Trainer™) under the desk tracked pedalling behaviours, and transmitted data over Bluetooth to an iPod touch with a third-party App installed to provide real-time feedback on pedal time, speed, distance, caloric expenditure<br>ID: informational and motivational emails promoting improved posture, regular breaks from sitting, self-efficacy for physical activity, small changes to the work environment and tips for reducing occupational stress<br>PDC: ankle-worn accelerometer (GENEActiv Original)	RCT (I (integrated health protection/health promotion) =27; C (health protection–only) = 27) + descriptive quantitative/ process evaluation (monitoring data + survey)	**User-related:** I group increased occupational light intensity PA relative to C; adherence and improvements in several cardio metabolic biomarkers (weight, total fat mass, resting heart rate, body fat percentage) and work productivity outcomes (concentration at work, days missed because of health problems)<br>**Design-related:** I group used the activity permissive workstations 70% of all intervention working days and 50 minutes/work day. the ergonomic assessment rated as most helpful, followed by regular emails.
(Donath *et al.*, 2015)	SP: some desktop software popped up dismissible prompts for sit-to-stand transitions at fixed times 3 times /day<br>PDC: ActiGraph for outcome measurement (for 1 week at baseline, 6-week and 12-week follow-up)	RCT<br>(I =15; C (no prompt, SSD-only) = 16)<br>(1-week baseline +12-week intervention)	**User-related:** n.s. change in sitting/standing or n.s. group × time interactions
(Evans *et al.*, 2012)	SP: desktop software (MyRestBreak 1.0) delivers non-dismissible prompts for breaks every 30 min<br>PDC: activPAL for outcome measurement (throughout the study period)	RCT (I=14; C (no prompt, education only) =14)<br>(1-week baseline + 1-week intervention)	**User-related:** Sig. reductions in sitting >=30 min (-1.1 events/workday, -60 min/workday) relative to control; n.s. difference in total sitting.
(Swartz *et al.*, 2014)	SP: desktop software (TimeLeft), and wrist-worn beeping device (Armitron MD0346-R(T)-2) or vibrating device (WobL Watch) delivered dismissible prompts for standing up (I_1) or standing up and walk for at least 100 steps (I_2) every 60 min<br>PDC: activPAL for outcome measurement (for 3 consecutive workdays in the baseline and intervention week);<br>PDC & ATF: Yamax SW-200 pedometer for real-time feedback on step count (I_2)	RCT (I_1(prompt to stand) =29, I_2 (prompt to take steps) =31)<br>(1-week baseline + 1-week intervention)	**User-related:** I_2 sig. reduced avg. duration of sitting bouts and number of sitting >=60 min, but not total sitting; I_1 sig. reduced total sitting (-25 min/workday), longest sitting, number of sitting>=30 min, increased sit-to-stand transitions
(Júdice *et al.*, 2015)	ID & SP: desktop software (Workrave,) delivers non-dismissible prompts every 60 min to take a 7-min walk<br>PDC: activPAL and ActiGraph GT3X+ for outcome measurement (for 21 days in total, throughout baseline, control and intervention periods);<br>PDC & ATF: OMRON pedometer real-time feedback on step count	Randomised crossover (n=10) (1-week baseline + 1-week washout + 1-week control + 1-week intervention with randomised order) + interviews + monitoring data	**User-related:** activPAL captured a sig. reduction in total sitting (-111 min/day) and n.s. increase in sit/stand transitions; ActiGraph captured n.s. change in number of breaks<br>**Design-related:** Interviews and behavioural data suggested people were resistant to increasing breaks, even though they reduced total sitting; 6 of the 10 participants were extremely satisfied with the program; 10 rated step goals as the best strategy (other than screen-based prompts and personally delivered behavioural strategies) to achieve sitting-time reduction; 7 reported leisure-time to be the greatest domain for reducing siting-time.

(Cooley and Pedersen, 2013)	ID & SP: desktop software (Exertime) delivers non-dismissible prompts for breaks and suggests exercises every 45 min for the first 13 weeks; for control period, the participants could voluntarily engage the software<br>DL & ATF: manually enter break activities in the software which provide visual feedback on exercise progress	Descriptive quantitative (13-week intervention + control 13-week period) (n=46)	**Design-related:** employees were willing to participate in a coercive workplace e-health intervention.<br>**User-related:** more activities per day and higher odds of (proxy) compliance (recording more than 6 activities per day) were recorded in passive prompt condition than active condition.
(Cooley, Pedersen and Mainsbridge, 2014)		Qualitative (Interview with 15 out of the 46 participants in a trial)	**Design-related outcomes:** 44 refused to uninstalling the software at the end of study; frustration and difficulty in adjusting to a new work behaviour and annoyance with the passive prompt; although some eventually adapted the way of working, proved to be resilient and accommodating<br>**User-related outcomes:** associated concept of exercising with enjoy and freedom; the tech promoted a sense of discovery; changed perception of what constituted exercise; the changed the environment to afford behaviour change; affected by state-wide competition; personalised feedback supported goal-setting and enhanced motivation; increased awareness and modified behaviour consciously to counteract habitual responses; the software stimulated more social interaction and communication between colleagues and changed the workplace climate.
(Pedersen, Cooley and Mainsbridge,		RCT<br>(I=17, C (maintain) =17)<br>(13-week intervention)	**Design-related:** 100% adherence with no withdrawal throughout the 13-week intervention period<br>**User-related:** OSPAQ self-report captured a reduction in total sitting (-55 min/workday, p value unreported) relative to C group at 13-week follow-up
(Mainsbridge *et al.*, 2014)	Same as above except that C group did not have access to the software	RCT (I = 11; C = 18)<br>(13-week intervention)	**Design-related:** The e-health software prompted participants in the experimental group to engage in NEPA for 7.99± 4.44 minutes per day by performing short-burst physical activities 6.28 ± 3.59 times per workday. The average time for each bout of NEPA was 1.34 ± 0.74 minutes.<br>**User-related:** Sig. decrease in mean arterial pressure in the experimental group relative to control.

(Taylor *et al.*, 2016)	ID & SP (I_2 only): computer software (Workrave, Eyes Relax, and Compact Timer) delivered 5 hourly prompts for 3-min breaks suggesting the user get up and walk hallways, stairs, or outdoors; user can skip or postpone the break.<br>PDC: Yamax SW200 pedometer for outcome measurement (1 week at baseline and 6-month follow-up)<br>DL: user completed daily log on indicating whether they ignored, partially met, or fully met the computer-prompted physical activity breaks	RCT (cluster) (I_1(Booster Break, full programme) = 69; I_2 (Computer Prompts only) = 59; C= 47)	**User-related:** varied results across groups; but overall speaking, consistent attendees of I_1 was observed with a sig. decrease in self-report weekday SB and increase in weekly pedometer counts and weekly energy expenditure.<br>**Design-related (Unintended):** I_2 showed increased self-report computer use
(Green, Sigurdsson and Wilder, 2016)	PDC: ActiGraph GT3X+ for outcome measurement<br>SP: WatchMinder 3 delivered tactile prompt and displayed text every 30 min, independent of movement.<br>ID: daily email with researcher-curated feedback on prolonged sitting bouts accumulated on the previous workday	Pre-post (n=3)	**User-related:** the tactile prompt plus feedback and goal setting phase was most effective for all participants in reducing the number and duration of prolonged sitting bouts (both >31 and >60).<br>**Design-related:** WatchMinder was worn 89% of those days the researcher randomly visited participants; 100%,
(Mackenzie, Goyder and Eves, 2015)	ID: weekly emails containing educational video, links to reminder software, and tips on sitting reduction and social media to increase awareness;<br>SP: choice to use a break reminder software<br>ID & MOSSI: encouragement emails from management	Pre-post (n=17)	**User-related:** Sitting reduced by 26 min/workday post-intervention from baseline based on sitting log.<br>**Design-related:** intervention was perceived as generally acceptable and feasible for the work context; mixed views of standing/walking meetings; some reported software as useful to keep the idea in consciousness and give ideas about things to try, whereas others reported as distracting. Only 38.5% were aware of this component.
(Bond *et al.*, 2014)	PDC & ATF & SP (JITAI): B-Mobile: Android smartphone accelerometer automatically monitored SB, the data about which was accessed in real-time by the researcher; audible prompt with on-screen text reminder of a 3-min break every 30 min; 6-min every 60 min; 12-min every 120 min; the user had the option to silence or delay the prompt for 30 min; a praising message and "go" badge will appear to reward compliance with prompts and the 'fuel gauge' will be refilled;<br>PDC: SenseWear Mini Armband for outcome measurement based on a combination of movement and physiological sensing data;<br>(throughout the baseline week and 3-week intervention period (1week for each condition)	Randomised crossover design (1-week for each condition) (n=30)	**User-related:** sitting reduced by 47.2 min/day (5.9%), 44.5 min/day (5.6%) and 26.2 min/day (3.2%) in 3-min, 6-min and 12-min conditions respectively relative to baseline;<br>**Design-related:** real-time display and feedback increased motivation to take breaks; siting reduced decreased as a result of the ID and ATF; decrease in SB as a result of using B-mobile; 6-min (n =17) condition as the most preferred condition, followed by 3-min (n=10) and 12-min conditions (n =3)
(Thomas and Bond, 2015)		Descriptive quantitative (use monitoring data to examine behavioural responses)	**Design-related:** the 3- and 6-min conditions resulted in the greatest number and sum duration of walking breaks, the best and fastest adherence to prompts; number and duration of breaks and adherence decreased significantly as a function of days accrued within a condition; high average latency between prompts and break start; 40.9%, 41.4%, and 33.8% of walking breaks were initiated within 5 minutes of the prompt in the 3-min, 6-min, and 12-min conditions, respectively. Conclusion: frequent prompts for small change may be an optimal strategy.

(He and Agu, 2014)	PDC & ATF & SP (JITAI): On11 – An Android app using smartphone accelerometer to classify activities into running, walking, sitting and 'phone on table'; main App screen showed daily activity summary including minutes spent sitting, walking and running; if the user was inactive (sitting or 'phone on table') for 27 minutes in the past 30 minutes, it flashed an LED light on the phone, triggered a vibration or sound, and used a push notification to ask the user to stand up and take a walk	Design & development (2-week field study + open-ended questionnaire; n= 8)	**Design-related: Feasibility -** five found the (in)activity detection accurate when phone was attached to body; long periods of 'phone on table'; battery drained quickly. **Acceptability -** 'at-a-glance' presentation of daily summary was liked; annoyance with the SP - didn't want to be bothered by telling them 'they were inactive' and to take a detour walk too frequently even if they knew it was true; could be encouraged if there was reward and low effort involved.
(Mohadis and Ali, 2016)	PDC & SP (JITAI): WargaFit - A smartphone App reminded users (older office workers) to do stretching after hours of sedentary office work<br>PDC & ATF & MOSSI: the App monitored step counts, provided feedback on the health outcomes of their physical activity and allowed social comparison with colleagues	Design & development (lab sessions with think-aloud protocol, participatory design, parallel prototyping; n=8)	**Design-related:** the system should have contextual awareness, visualise how behaviours would impact on health status, present data that are accurate and reliable, and give user the control over the collection and sharing of their personal health information with colleagues.
(Mukhtar and Belaid, 2013)	PDC & SP (JITAI): SedentaWare - using Android API for activity recognition; if the user had been inactive for > 50% of the last 2 hours, a notification was launched on phone and the threshold was incremented by 10% so that the next reminder would be triggered after being inactive for > 60% in the last 2 hours.<br>PDC & ATF: activity dashboard showed inactive minutes since last activity and reminder countdown; daily, weekly and monthly comparison on activity/inactivity; colour coded icon indicating the need for activity remained all the time visible on the phone's notification bar	Design & development (3-day baseline + 1-week field study, n=4, 2 students and 2 faculty members)	**Design-related:** reminders with adaptive thresholds were more favourably received by users.<br>**User-related:** significant decrease in % of inactivity from baseline after introducing the final version of SedentaWare;
(Grundgeiger *et al.*, 2017)	PDC & SP (JITAI): An Android App combined the gyroscope, light sensors and an existing step counter to detect sitting/standing up/walking; if the user had <2 MET-minutes activity in 30 minutes, the phone vibrated to remind the user to get active, which was repeated once after 90 seconds; if the user was active before 30 min, timers were reset and the user received a silent positive notification.	Design &development (1-day baseline +2-day static reminder + 2-day dynamic App, exit survey, n=5)	**Design-related:** receiving positive notifications from the dynamic App more frequently than from the static App. Sig. difference between 85% subjective and 14% objective compliance rate highlights the need for objective activity tracking measures.<br>**User-related:** significantly less sitting and more MET with the App than at the baseline

(Wadhwa *et al.*, 2015)	PDC & ATF & MOSSI : *SenseX* mobile service captured activities and contextual attributes with smartphone sensors; the App *Standup* visualised daily activities along a timeline, provided feedback on weekly performance, and organisation leaderboard.<br>PDC & ATF: SenseX workstation service tracked keyboard, mouse activities and webcam-based feed and visualised such activity levels.<br>PDC & SP (JITAI): both workstation and mobile services provided onscreen notifications for breaks<br>PDC & CD: used triggered-sensing to replace some mobile sensing with infrastructure sensing and improve battery life; perform sensor fusion for activity inference	Design & development, Descriptive quantitative (8 weeks, n=30)	**Design-related:** mobile and workstation services sensed nearly 50% and 25% of the total time respectively; triggered-sensing could increase the battery life time by nearly 25% of the time. Based on response time, there was a slight preference for mobile-based notifications over workstations ones; long tail of longer response times was observed because participants did not carry their mobile phones when they took a break.<br>**User-related:** increased step counts and recued sitting sessions/durations were observed from week 1 to week 6 (no baseline info); for nearly half of the notifications, participants acted in less than 10 minutes.
(Van Dantzig, Geleijnse and Van Halteren, 2013)	Study 1<br>PDC & SP (JITAI): an Android App (SitCoach) using on-board accelerometer of smartphone to track inactive minutes; the phone vibrated or buzzed after a configurable amount of inactive minutes (60 min by default)<br>PDC & ATF: the App displayed accumulative daily active minutes and countdown until next break	Design & development (1-day use of App + interviews, n =8)	**Design-related outcomes:** tactile feedback is preferred to acoustic alert; main barrier to break is perceived lack of control over their sedentary breaks, which was also source of annoyance with PC break reminders; smartphone battery drains quickly with accelerometer running
	Study 2<br>PDC: software tracking mouse and keyboard movements for outcome measurement; commercial monitor (brand unknown) for PA outcome measurement (baseline week and 6-week intervention period)<br>PDC & CD & SP (JITAI): mouse and keyboard movement was tracked and uploaded to a backend server which sent text messages to phone containing hyperlinks to persuasive messages for breaks in every 30 minutes of computer activity<br>PDC & CD & ATF: commercial PA monitor data could be uploaded to the computer via USB cable and a personal webpage for feedback on activity pattern	RCT (I=40; C (no break prompts) =46))	**User-related outcomes:** message leads to Steeper post-message decrease in computer activity in intervention group than control; seems to have post-message peak in PA, but n.s. difference from control;<br>**Design-related outcomes:** no larger effect in those reading > 50% of messages, which suggests that the content of the persuasive messages is unimportant, receiving a timely reminder on their smartphone might be sufficient)
(Davis *et al.*, 2009)	PDC & SP (JITAI): Workpace™ software monitored key strokes and mouse movement, work intensity and compliance to break (e.g. no keyboard or mouse activity), reminder software delivered dismissible prompts every 30 min, participants had the freedom to follow or ignore prompts.<br>PDC: video camera were set up for outcome measurement (in the last 2 weeks of each condition)	Randomised crossover (each of the four conditions was evaluated for 1 month; n=35)	**User-related:** reminder software had the largest impact on discomfort reduction and even increased productivity

(Ferreira *et al.*, 2014)	PDC & CD & SP (JITAI): A desktop system (BreakOut) used webcam to track posture, keyboard/mouse sensor to track computer activity delivered just-in-time prompts via a sculpture built on the Arduino platform<br>ID: using desktop wallpapers and a sculpture to display information on need of a break	4-day field study with validation data from Experience Sampling (n = 10)	Sig. correlation between self-report and inferred posture, stress and engagement; posture and stress, but not engagement predicts need of a break.
(Van Almkerk *et al.*, 2015)	PDC & CD & ATF: Backtive - a chair (built on Arduino) with embedded push buttons and pressure sensor capturing pressure distribution; data was transmitted to a laptop with a cable; data was then sent to smartphone application that provided visual feedback on daily sitting time and posture.<br>PDC & ATF & SP (JITAI): chair provides tactile feedback (pumped air cushion at the back) for just-in-time posture correction<br>DL: user input the amount of pain experienced in the smartphone application	Design & development (2-hour lab testing + questionnaire; n=4)	**Design-related:** survey suggested participants had high behavioural intention to use Backtive, expected it to be easy to use; there is room for aesthetic improvement
(Gilson *et al.*, 2016)	PDC: Sitting Pads with pressure sensors developed by the University for outcome measurement (baseline week + 5-month intervention); GENEActiv wrist accelerometer (baseline week & the last week in the intervention period)<br>I_2 Group Only:<br>PDC & CD & ATF: proprietary software that linked to the Sitting Pads to download data and summarised duration of total sitting and longest sitting bout<br>PDC & CD & SP (JITAI): software prompts moved from Green to Amber and then Red repetitively after 30 min and then 60 min of continuous sitting (user can tailor the time threshold and opt-in for additional auditory prompts)	Pre-post with comparison group (I_1 (participatory only, no prompt)= 33, I_2 (prompt + participatory approach) = 24)	**User-related:** GENEActiv captured sig. sitting reduction (-54 min/day) in I_2 relative to I_1.<br>Based on Sitting Pads data, there was sig. reduction in total sitting (-23 min/day) and longest bout sitting (-32 min/day) from baseline in I_2 relative to I_1.
(Jafarinaimi *et al.*, 2005)	PDC & CD & SP (JITAI): a prototype system built on the Parallax Basic Stamp Board of Education included a chair cushion with conductive sensor that captured sitting time and a sculpture with servo motor that changed its shape after 60 and 90 minutes of sitting to prompt standing.<br>ID: sculpture delivered information on the harm of prolonged sitting	Design & development (interview + baseline week + intervention week + post-study interview, n=1)	**Design-related:** Demonstrate its technological **feasibility** and **acceptability**; users appreciate the non-intrusiveness and aesthetics.<br>**Behavioural outcome:** there is relationship between the sculpture movement and user's break times. Long-term experiment is needed.

(Haller *et al.*, 2013)	PDC & CD & SP & ATF (JITAI): a prototype of intelligent chairs that classified sitting into 8 postures based on four force transducers, was connected to three options of devices for three types of feedback: 1) graphical: onscreen digital prompts for exercise; 2) physical: mechanically controlled physical plants; 3)vibrotactile: vibrations and buzzes	Lab study with within-subject design (1.5 hours for all conditions, n=12)	**Design-related:** Less postponed responses to prompts in editing task than in transcription and planning tasks; vibrotactile feedback led to shortest response time than other feedback, but was rated as the most disturbing form of ongoing feedback. *Physical feedback* was rated as least disruptive to workflow, least disturbing form for providing ongoing feedback/alerts, and also required the shortest time to return to the main task after the prompted activity; time to return to the primary task was longest under the digital feedback condition.
(El-sayed *et al.*, 2011)	PDC & CD & SP (JITAI): a bulky wearable prototype system that consisted of weight sensors in shoes measured the strain placed on each foot to detect prolonged sitting and standing in fixed positions, inclinometer at the neck measured bending angle of upper back, a WiFi device transmitted sensor data to a base station. The system sent SMS message to user when sustained bad posture was detected<br>ATF: desktop interface provided real-time feedback on posture and daily time spent standing, sitting or walking; summary report was also sent by email at the end of the day.	Design & development (Lab testing; N unknown)	**Design-related:** accurate detection of both poor back posture and prolonged sitting/standing (technological feasibility); bulky wearable components need to be downsized (practical issue)
(Fortmann *et al.*, 2013)	PDC & CD & SP & ATF (JITAI): MoveLamp system - an Android smartphone with pedometer application transmitted step counts to a desktop application via Bluetooth; desktop app calculated colour value and manipulated the colour of an ambient light display via radio transmission; bright red signified 2 hours of no steps and suggested the need for a break; 500 steps would restore it to dark green.<br>PDC: pedometer data for outcome measurement	Randomised crossover (1-day control (pedometer App only) + 1-day intervention; n=10)	**User-related:** ambient light feedback on inactivity helped people to both move more frequently and increased overall daily steps.
(Mateevitsi *et al.*, 2014)	PDC & CD & ATF & SP (JITAI): HealthBar – An Arduino controlled light tube changes colour from green to red, corresponding to the length of the user's sitting period, which was captured as presence at desk by passive infrared motion sensors; after 45 min of sitting, the tube will pulse twice every 5 min to prompt a break in sitting; the bar can be reset by taking a 5 -min break.	Design & development (5-day field study + pre- and post-study questionnaire; n=8)	**Design-related:** All agreed the participants was easy to understand and make sense; all would like to continue using the system; perceived as "helpful" and "interesting".<br>**User-related:** 4 thought it was a light distraction, 3 thought it was not a distraction; majority reported increased awareness of unhealthy sitting habits and breaks as a result of using the system
(Reeder *et al.*, 2010)	PDC & SP (JITAI) (scenario and concept): After 45 minutes of sitting, the Arduino-based Breakbot' fibre optic ear tufts blinked red and retracted into its head to express displease and tiredness; vibrated after another 15min of inactivity; and waved tufts to show happiness if the user took a break.<br>IP: use animated features to express robot's emotions	Design & development (interview(n=6), diary(n=3), survey (n=16))	**Design-related:** Preferred form of robots spanned the range of minimal to creature-like; the minimal form of prototype was met with enthusiasm, though size and portability was a concern; personalisation would be valuable; showed emotional investment in the robot's welfare.

(Obermair *et al.*, 2008)	PDC (Wizard of Oz) & SP (JITAI): perFrame – researcher observed user's sitting posture via a camera and controlled an electronic photo frame via WiFi to display selected video footages<br>ID & MOSSI: in the videos, either an actor or the user's close friend perform gestures and expressions to show approval/disapproval for the user's sitting behaviours.	Design & development (half a day + interview, n=8)	**Design-related:** users rated the interface as pleasing and useful; but could not always comprehend the meaning of the portraits of a unfamiliar actor; high likability and comprehensibility of a close friend's videos compared with an actor's; there was also a lack of consistency in standard for good vs. bad posture; when the portrait performed a gesture, they noticed; it was not distracting and glimpses were voluntary

I: Intervention
C: control or comparison

Reference:

Aittasalo, M. *et al.* (2017) 'Moving to business – changes in physical activity and sedentary behavior after multilevel intervention in small and medium-size workplaces', *BMC Public Health*. BMC Public Health, 17(1), p. 319. doi: 10.1186/s12889-017-4229-4.

Van Almkerk, M. *et al.* (2015) 'Improving posture and sitting behavior through tactile and visual feedback in a sedentary environment', *Proceedings of the Annual International Conference of the IEEE Engineering in Medicine and Biology Society, EMBS*, 2015–Novem, pp. 4570–4573. doi: 10.1109/EMBC.2015.7319411.

van Berkel, J. *et al.* (2011) 'Mindful "Vitality in Practice": an intervention to improve the work engagement and energy balance among workers; the development and design of the randomised controlled trial', *BMC Public Health*. BioMed Central Ltd, 11(1), p. 736. doi: 10.1186/1471-2458-11-736.

van Berkel, J. *et al.* (2014) 'Effectiveness of a worksite mindfulness-based multi-component intervention on lifestyle behaviors.', *The international journal of behavioral nutrition and physical activity*, 11, p. 9. doi: 10.1186/1479-5868-11-9.

Bond, D. S. *et al.* (2014) 'B-MOBILE - A smartphone-based intervention to reduce sedentary time in overweight/obese individuals: A within-subjects experimental trial', *PLoS ONE*, 9(6). doi: 10.1371/journal.pone.0100821.

Bort-Roig, J. *et al.* (2014) 'Uptake and factors that influence the use of `sit less, move more¿ occupational intervention strategies in Spanish office employees.', *The international journal of behavioral nutrition and physical activity*, 11(1), p. 152. doi: 10.1186/s12966-014-0152-6.

Brakenridge, C. L. *et al.* (2016) 'Evaluating the effectiveness of organisational-level strategies with or without an activity tracker to reduce office workers' sitting time: a cluster-randomised trial.', *The international journal of behavioral nutrition and physical activity*. International Journal of Behavioral Nutrition and Physical Activity, 13(1), p. 115. doi: 10.1186/s12966-016-0441-3.

Carr, L. J. *et al.* (2013) 'Multicomponent intervention to reduce daily sedentary time: a randomised controlled trial.', *BMJ open*, 3(10), p. e003261. doi: 10.1136/bmjopen-2013-003261.

Carr, L. J. *et al.* (2015) 'Total Worker Health Intervention Increases Activity of Sedentary Workers.', *American journal of preventive medicine*, 50(1), pp. 9–17. doi: 10.1016/j.amepre.2015.06.022.

Carr, L. J., Walaska, K. A. and Marcus, B. H. (2012) 'Feasibility of a portable pedal exercise machine for reducing sedentary time in the workplace', *British Journal of Sports Medicine*, 46(6), pp. 430–435. doi: 10.1136/bjsm.2010.079574.

De Cocker, K. *et al.* (2015) 'Theory-driven, web-based, computer-tailored advice to reduce and interrupt sitting at work: development, feasibility and acceptability testing among employees', *BMC Public Health*. BMC Public Health, 15(1), p. 959. doi: 10.1186/s12889-015-2288-y.

De Cocker, K. *et al.* (2016) 'The Effectiveness of a Web-Based Computer-Tailored Intervention on Workplace Sitting: A Randomized Controlled Trial', *Journal of Medical Internet Research*, 18(5). doi: 10.2196/jmir.5266.
Coffeng, J. K. *et al.* (2012) 'The development of the Be Active & Relax "Vitality in Practice" (VIP) project and design of an RCT to reduce the need for recovery in office employees', *BMC Public Health*, 12(1), p. 592. doi: 10.1186/1471-2458-12-592.
Coffeng, J. K. *et al.* (2013) 'Process evaluation of a worksite social and physical environmental intervention.', *Journal of occupational and environmental medicine / American College of Occupational and Environmental Medicine*, 55(12), pp. 1409–20. doi: 10.1097/JOM.0b013e3182a50053.
Coffeng, J. K. *et al.* (2014) 'Effectiveness of a worksite social & physical environment intervention on need for recovery, physical activity and relaxation; results of a randomized controlled trial', *PLoS ONE*, 9(12), pp. 1–26. doi: 10.1371/journal.pone.0114860.
Compernolle, S. *et al.* (2015) 'Effectiveness of a web-based, computer-tailored, pedometer-based physical activity intervention for adults: A cluster randomized controlled trial', *Journal of Medical Internet Research*, 17(2), pp. 1–17. doi: 10.2196/jmir.3402.
Cooley, D. and Pedersen, S. (2013) 'A pilot study of increasing nonpurposeful movement breaks at work as a means of reducing prolonged sitting', *Journal of Environmental and Public Health*, 2013. doi: 10.1155/2013/128376.
Cooley, D., Pedersen, S. and Mainsbridge, C. (2014) 'Assessment of the impact of a workplace intervention to reduce prolonged occupational sitting time.', *Qualitative health research*, 24(1), pp. 90–101. doi: 10.1177/1049732313513503.
Van Dantzig, S., Geleijnse, G. and Van Halteren, A. T. (2013) 'Toward a persuasive mobile application to reduce sedentary behavior', *Personal and Ubiquitous Computing*, 17(6), pp. 1237–1246. doi: 10.1007/s00779-012-0588-0.
Davis, K. G. *et al.* (2009) 'Combating the Effects of Sedentary Work : Postural Variability Reduces Musculoskeletal Discomfort', pp. 884–886.
Donath, L. *et al.* (2015) 'Repetitive daily point of choice prompts and occupational sit-stand transfers, concentration and neuromuscular performance in office workers: an RCT.', *International journal of environmental research and public health*. Multidisciplinary Digital Publishing Institute, 12(4), pp. 4340–53. doi: 10.3390/ijerph120404340.
El-sayed, B. *et al.* (2011) 'A Novel Mobile Wireless Sensing System for Real- time Monitoring of Posture and Spine Stress', in *1st Middle East Conference on Biomedical Engineering*, pp. 428–431.
Evans, R. E. *et al.* (2012) 'Point-of-choice prompts to reduce sitting time at work: a randomized trial.', *American journal of preventive medicine*, 43(3), pp. 293–7. doi: 10.1016/j.amepre.2012.05.010.
Ferreira, M. J. *et al.* (2014) 'Breakout: predicting and breaking sedentary behaviour at work', *Proceedings of the extended abstracts of the 32nd annual ACM conference on Human factors in computing systems - CHI EA '14*, pp. 2407–2412. doi: 10.1145/2559206.2581330.
Fortmann, J. *et al.* (2013) 'Make Me Move at Work ! An Ambient Light Display to Increase Physical Activity', in *PervasiveHealth*, pp. 3–6.
Freak-Poli, R. *et al.* (2011) 'Impact of a pedometer-based workplace health program on cardiovascular and diabetes risk profile', *Preventive Medicine*. Elsevier Inc., 53(3), pp. 162–171. doi: 10.1016/j.ypmed.2011.06.005.
Freak-Poli, R. *et al.* (2013) 'Eight-month postprogram completion: Change in risk factors for chronic disease amongst participants in a 4-month pedometer-based workplace health program', *Obesity*, 21(9), pp. 360–368. doi: 10.1002/oby.20342.
Freak-Poli, R. *et al.* (2014) 'Change in well-being amongst participants in a four-month pedometer-based workplace health program', *BMC Public Health*, 14(1), p. 953. doi: 10.1186/1471-2458-14-953.
Freak-Poli, R. LA *et al.* (2011) 'Participant characteristics associated with greater reductions in waist circumference during a four-month, pedometer-based, workplace health program', *BMC Public Health*, 11(1), p. 824. doi: 10.1186/1471-2458-11-824.
Ganesan, A. N. *et al.* (2016) 'International Mobile-Health Intervention on Physical Activity, Sitting, and Weight: The Stepathlon Cardiovascular Health Study', *Journal of the American College of Cardiology*, 67(21), pp. 2453–2463. doi: 10.1016/j.jacc.2016.03.472.

Gilson, N. *et al.* (2007) ‘Walking towards health in a university community: A feasibility study’, *Preventive Medicine*, 44(2), pp. 167–169. doi: 10.1016/j.ypmed.2006.09.012.
Gilson, N. D. *et al.* (2009) ‘Do walking strategies to increase physical activity reduce reported sitting in workplaces: a randomized control trial.’, *The international journal of behavioral nutrition and physical activity*, 6, p. 43. doi: 10.1186/1479-5868-6-43.
Gilson, N. D. *et al.* (2016) ‘Project Energise: Using participatory approaches and real time computer prompts to reduce occupational sitting and increase work time physical activity in office workers’, *Journal of Science and Medicine in Sport*. Sports Medicine Australia, 19(11), pp. 926–930. doi: 10.1016/j.jsams.2016.01.009.
Gilson, N., McKenna, J. and Cooke, C. (2008) ‘Experiences of Route and Task-Based Walking in a University Community: Qualitative Perspectives in a Randomized Control Trial’, *Journal of Physical Activity & Health*, 5(February), pp. S176–S182. doi: 10.1123/jpah.5.s1.s176.
Green, N., Sigurdsson, S. and Wilder, D. A. (2016) ‘Decreasing bouts of prolonged sitting among office workers’, *Journal of Applied Behavior Analysis*, 49(3), pp. 717–722. doi: 10.1002/jaba.309.
Grundgeiger, T. *et al.* (2017) ‘Combating Sedentary Behavior : An App Based on a Distributed Prospective Memory Approach’, pp. 1632–1639. doi: 10.1145/3027063.3053094.
Haller, M. *et al.* (2013) ‘Finding the right way for interrupting people to posture guidance’, *Proceedings of the 13th IFIP TC 13 international conference on Human-computer interaction*, pp. 1–18.
He, Q. and Agu, E. (2014) ‘On11 : An Activity Recommendation Application to Mitigate Sedentary Lifestyle’, in *ACM Workshop on Physical Analytics, Co-located with MobiSys*, pp. 3–8.
Healy, G. *et al.* (2013) ‘Reducing sitting time in office workers: short-term efficacy of a multicomponent intervention.’, *Preventive medicine*, 57(1), pp. 43–8. doi: 10.1016/j.ypmed.2013.04.004.
Healy, G. N. *et al.* (2016) ‘A Cluster Randomized Controlled Trial to Reduce Office Workers’ Sitting Time: Effect on Activity Outcomes’, *Medicine and Science in Sports and Exercise*, 48(9), pp. 1787–1797. doi: 10.1249/MSS.0000000000000972.
J. Carr, L. *et al.* (2014) ‘Acceptability and effects of a seated active workstation during sedentary work’, *International Journal of Workplace Health Management*, 7(1), pp. 2–15. doi: 10.1108/IJWHM-03-2013-0008.
Jafarinaimi, N. *et al.* (2005) ‘Breakaway : An Ambient Display Designed to Change Human Behavior’, in *CHI’05 extended abstracts on Human factors in computing systems*, pp. 1945–1948. doi: 10.1145/1056808.1057063.
Júdice, P. B. *et al.* (2015) ‘Randomized controlled pilot of an intervention to reduce and break-up overweight/obese adults’ overall sitting-time.’, *Trials*. Trials, 16, p. 490. doi: 10.1186/s13063-015-1015-4.
Kerr, J. *et al.* (2016) ‘Two-arm randomized pilot intervention trial to decrease sitting time and increase sit-to-stand transitions in working and non-working older adults’, *PLoS ONE*, 11(1), pp. 1–12. doi: 10.1371/journal.pone.0145427.
Mackenzie, K., Goyder, E. and Eves, F. (2015) ‘Acceptability and feasibility of a low-cost, theory-based and co-produced intervention to reduce workplace sitting time in desk-based university employees’, *BMC Public Health*. BMC Public Health, 15(1), p. 1294. doi: 10.1186/s12889-015-2635-z.
MacNiven, R. *et al.* (2015) ‘Does a corporate worksite physical activity program reach those who are inactive? Findings from an evaluation of the Global Corporate Challenge’, *Health Promotion Journal of Australia*, 26(2), pp. 142–145. doi: 10.1071/HE14033.
Mainsbridge, C. P. *et al.* (2014) ‘The Effect of an e-Health Intervention Designed to Reduce Prolonged Occupational Sitting on Mean Arterial Pressure’, *Journal of Occupational and Environmental Medicine*, 56(11), pp. 1189–1194. doi: 10.1097/JOM.0000000000000243.
Marshall, A. L. *et al.* (2003) ‘Print versus website physical activity programs: a randomized trial’, *American Journal of Preventive Medicine*. Elsevier, 25(2), pp. 88–94. doi: 10.1016/S0749-3797(03)00111-9.

Mateevitsi, V. *et al.* (2014) ‘The Health Bar : A Persuasive Ambient Display to improve the office worker’s well being’, *Proceedings of the 5th Augmented Human International Conference*, p. 21. doi: 10.1145/2582051.2582072.
Mohadis, H. M. and Ali, N. M. (2016) ‘Designing persuasive application to encourage physical activity at workplace among older workers’, *2016 6th International Conference on Digital Information and Communication Technology and Its Applications, DICTAP 2016*, pp. 126–130. doi: 10.1109/DICTAP.2016.7544013.
Mukhtar, H. and Belaid, D. (2013) ‘Using adaptive feedback for promoting awareness about physical activeness in adults’, *Proceedings - IEEE 10th International Conference on Ubiquitous Intelligence and Computing, UIC 2013 and IEEE 10th International Conference on Autonomic and Trusted Computing, ATC 2013*, pp. 638–643. doi: 10.1109/UIC-ATC.2013.99.
Neuhaus, M., Healy, G. N., *et al.* (2014) ‘Iterative development of Stand Up Australia: a multi-component intervention to reduce workplace sitting.’, *The international journal of behavioral nutrition and physical activity*, 11(1), p. 21. doi: 10.1186/1479-5868-11-21.
Neuhaus, M., Healy, G. N., *et al.* (2014) ‘Workplace sitting and height-adjustable workstations: A randomized controlled trial’, *American Journal of Preventive Medicine*, 46(1), pp. 30–40. doi: 10.1016/j.amepre.2013.09.009.
Obermair, C. *et al.* (2008) ‘PerFrames: Persuasive picture frames for proper posture’, *Lecture Notes in Computer Science (including subseries Lecture Notes in Artificial Intelligence and Lecture Notes in Bioinformatics)*, 5033 LNCS, pp. 128–139. doi: 10.1007/978-3-540-68504-3-12.
Parry, S. *et al.* (2013) ‘Participatory workplace interventions can reduce sedentary time for office workers--a randomised controlled trial.’, *PloS one*, 8(11), p. e78957. doi: 10.1371/journal.pone.0078957.
Pedersen, S. J., Cooley, P. D. and Mainsbridge, C. (2014) ‘An e-health intervention designed to increase workday energy expenditure by reducing prolonged occupational sitting habits’, *Work*, 49(2), pp. 289–295. doi: 10.3233/WOR-131644.
Priebe, C. S. and Spink, K. S. (2015) ‘Less sitting and more moving in the office: Using descriptive norm messages to decrease sedentary behavior and increase light physical activity at work’, *Psychology of Sport and Exercise*. Elsevier Ltd, 19, pp. 76–84. doi: 10.1016/j.psychsport.2015.02.008.
Puig-Ribera, A. *et al.* (2015) ‘Patterns of impact resulting from a “sit less, move more” web-based program in sedentary office employees’, *PLoS ONE*. Edited by H. Zhang. Wolters Kluver/Lippincott Williams & Wilkings, 10(4), p. e0122474. doi: 10.1371/journal.pone.0122474.
Puig-Ribera, A. *et al.* (2017) ‘Impact of a workplace “sit less, move more” program on efficiency-related outcomes of office employees’, *BMC Public Health*. BMC Public Health, 17(1), p. 455. doi: 10.1186/s12889-017-4367-8.
Rabbi, M. *et al.* (2015) ‘Automated personalized feedback for physical activity and dietary behavior change with mobile phones: a randomized controlled trial on adults.’, *JMIR mHealth and uHealth*, 3(2), p. e42. doi: 10.2196/mhealth.4160.
Reeder, S. *et al.* (2010) ‘Breakbot : A Social Motivator for the Workplace’, *Proceedings of the 8th ACM Conference on Designing Interactive Systems*, pp. 61–64. doi: 10.1145/1858171.1858184.
Slootmaker, S. M. *et al.* (2009) ‘Feasibility and effectiveness of online physical activity advice based on a personal activity monitor: randomized controlled trial.’, *Journal of medical Internet research*, 11(3), pp. 1–13. doi: 10.2196/jmir.1139.
Stephens, S. K. *et al.* (2014) ‘Intervening to reduce workplace sitting time: how and when do changes to sitting time occur?’, *British journal of sports medicine*, 48(13), pp. 1037–42. doi: 10.1136/bjsports-2014-093524.
Sternfeld, B. *et al.* (2009) ‘Improving Diet and Physical Activity with ALIVE. A Worksite Randomized Trial’, *American Journal of Preventive Medicine*. American Journal of Preventive Medicine, 36(6), pp. 475–483. doi: 10.1016/j.amepre.2009.01.036.
Swartz, A. M. *et al.* (2014) ‘Prompts to disrupt sitting time and increase physical activity at work, 2011-2012.’, *Preventing chronic disease*, 11, p. E73. doi: 10.5888/pcd11.130318.
Taylor, W. C. *et al.* (2016) ‘Impact of Booster Breaks and Computer Prompts on Physical Activity and Sedentary Behavior Among Desk-Based

Workers: A Cluster-Randomized Controlled Trial', *Preventing Chronic Disease*, 13(3), p. 160231. doi: 10.5888/pcd13.160231.
Thomas, J. G. and Bond, D. S. (2015) 'Behavioral response to a just-in-time adaptive intervention (JITAI) to reduce sedentary behavior in obese adults: Implications for JITAI optimization.', *Health psychology*, 34(0), pp. 1261–1267. doi: 10.1037/hea0000304.
Wadhwa, R. *et al.* (2015) 'SenseX: Design and deployment of a pervasive wellness monitoring platform for workplaces', *Lecture Notes in Computer Science (including subseries Lecture Notes in Artificial Intelligence and Lecture Notes in Bioinformatics)*, 9435, pp. 427–443. doi: 10.1007/978-3-662-48616-0_30.
